# Imaging findings of juvenile idiopathic arthritis and autoinflammatory diseases in children

**DOI:** 10.1007/s11604-023-01447-6

**Published:** 2023-06-17

**Authors:** Yuko Tsujioka, Gen Nishimura, Hideharu Sugimoto, Taiki Nozaki, Tatsuo Kono, Masahiro Jinzaki

**Affiliations:** 1 Department of Radiology, Keio University School of Medicine, 35, Shinanomachi, Shinjuku-Ku, Tokyo 160-0016 Japan; 2 Department of Radiology, Tokyo Metropolitan Children’s Medical Center, Tokyo, Japan; 3 Department of Radiology, Musashino-Yowakai Hospital, Tokyo, Japan; 4 Department of Radiology, Shin-Kaminokawa Hospital, Tochigi, Japan; 5 Department of Radiology, St. Luke’s International Hospital, Tokyo, Japan

**Keywords:** Juvenile idiopathic arthritis, JIA, Systemic JIA, Autoinflammatory disease, CRMO, Imaging findings of JIA

## Abstract

Juvenile idiopathic arthritis (JIA) is a collective term for pediatric inflammatory arthritis of unknown etiology, which presents diverse clinical and imaging findings. The pathogenesis is complex; however, most cases stem from an autoimmune mechanism. Herein we provide a short review of imaging findings of JIA. Imaging assessment begins with plain radiography demonstrating joint swelling, periarticular osteopenia, and juxtaarticular bone erosion. Bone erosion occurs later in JIA. Instead, aberrant epimetaphyseal growth often gives the first clue to the diagnosis. US and MRI can demonstrate the details of the synovium, cartilage, and subchondral bone. JIA is subdivided into oligoarthritis, polyarthritis (rheumatoid factor-negative and positive), psoriatic arthritis, enthesitis-related arthritis, and systemic JIA. Awareness of the different clinical characteristics, pathogenic background, and prognosis of each subtype facilitates a more advanced, imaging-based diagnosis. Unlike the other types, systemic JIA is an autoinflammatory disease accompanied by inflammatory cytokinemia and systemic symptoms stemming from aberrant activation of the innate immunity. Other autoinflammatory diseases, both monogenic (e.g., NOMID/CINCA) and multifactorial (e.g., CRMO), are also discussed.

## Introduction

Juvenile idiopathic arthritis (JIA), formerly termed juvenile rheumatoid arthritis or juvenile chronic arthritis, is the most common pediatric rheumatological disorder and has a prevalence of 16–150/100000 [1]. JIA is not a single disease, but encompasses a diverse group of chronic inflammatory arthritides. The disease definition is arthritis of unknown etiology (excluding known causes of joint inflammation) developing before age 16 years and persisting in one or more joints for at least 6 weeks. Its precise pathogenesis remains unknown. However, susceptibility to the disease is believed to be related to the patient’s genetic background and in the majority of cases synovial arthritis occurs as a result of a disturbance of the acquired immune system caused by an autoimmune mechanism that provokes aberrant cytokine production.

Early diagnosis and prompt treatment are essential for the management of children with JIA. In addition to the conventional anti-inflammatory agents (NSAIDs and corticosteroids), new medical interventions capable of suppressing inflammatory cytokines, such as disease-modifying anti-rheumatic drugs (DMARDs) and biological agents, may be able to improve outcomes in affected children. However, despite the recent progress in treatment, approximately half of affected children still experience some degree of physical disability as late sequelae of chronic synovitis.

The diagnosis of JIA is not necessarily straightforward. Contrary to the general belief and clinicians’ preconceptions, “inflammatory” findings are not always present as the initial symptoms. Inflammation in arthritis may be mild or insidious on clinical grounds. Therefore, the diagnosis is often delayed. The delayed diagnosis enhances a vicious cycle of cytokine activation and inflammatory response, further exacerbating the chronic inflammation. Imaging studies play a pivotal role in the early diagnosis of JIA by detecting pediatric-specific findings of chronic inflammation. They are also useful for monitoring the disease and evaluating the treatment response. As with adult rheumatoid arthritis (RA), radiological scoring has been used to evaluate the disease severity in JIA, but this method is not yet well established.

The present article provides a short review of the imaging findings and differential diagnoses of JIA. Also briefly discussed are autoinflammatory diseases caused by aberrant activation the innate, rather than the acquired immune system. It is now thought that unlike other types of JIA (joint-types of JIA), systemic JIA is caused by an autoinflammatory mechanism rather than being an autoimmune dysfunction [2, 3].

## Classification of JIA

Table 1 shows the currently used classification of JIA, namely the ILAR (International League of Associations for Rheumatology) classification. The clinical spectrum of JIA is more diverse than that of adult RA and probably includes conditions whose pathogenesis differs essentially from that of adult RA. JIA is classified into eight subgroups or disease subtypes, including five fundamental types, two variant types, and one undifferentiated type. The diagnostic criteria consist of the presence or absence of systemic symptoms, the number of affected joints within six months from onset (four or less; or five or more), and serological findings. The prognosis and complications differ among these groups; thus, a high level of skill is needed to understand the clinical and imaging findings characterizing each disease type. It is intriguing that the overall frequency of JIA and the distribution of the disease types differ among racial groups, suggesting a relationship between genetic background and JIA development.

**Table 1:** ILAR (International League of Associations for Rheumatology) classification of JIA

Subtipe	Peak age of onset *	Sex ratio *	Laboratory	Common features	Veitis risk *
Oligoarticular	2–4 years	F/M = 3:1	ANA + 70% (60%)	1–4 joints, often monoarticular Frequent knee, ankle, and wrist involvement Prolonged remission and good prognosis	Occcasionally (10%)
RF negative polyarticular	Bimodal, 2–4 years and 10–14 years	F/M = 3:1 and 10:1	ANA + 40–50% (40%)	> 5 joints Various pattterns of joint involvement and variable prognosis	Occcasionally (10%)
RF positive polyarticular	Adolescent	F/M = 9:1	ANA-	"Early-onset adult RA" Multiple symmetrical involvement of small joints Prolonged inflammation and progressive bone erosion Mlid systemic symptoms	Rare
Psoriatic arthritis	Bimodal, 2–4 years and 9–11 years	F/M = 2:1	ANA + (50%), HLA B-27 +	Various patterns of joint involvement Nail pitting, dactylitis, and family history of psoriasis Joint involvement often precedes psoriatic skin changes	Occcasionally (10%)
Enthesitis-related arthritis	9–12 years	F/M = 1:7	HLA B-27 + (80%)	Common pathogenesis with adult ankylosing spondylitis Asymmetric involvement of the large joints Enthesitis of the lower extremities At risk for later development of axial lesions	Acute uveitis; occasionally
Systemic	1–5 years	F/M = 1:1	ANA-	Common pathogenesis with adult-onset Still's disease Excessive production of inflammatory cytokines Various patterns of arthritis Severe joint destruction in about 1/4 of patients	Rare
Undifferentiated	Variable	–	–	–	–

*Nelson WE, Kliegman R. Nelson textbook of pediatrics. 21st ed. ed: Elsevier; 2020. 2 v. p.

The classification of JIA can broadly be divided into the systemic type and non-systemic (joint) types. Contrary to the previous understanding, “systemic JIA” is now considered to have a pathological background that differs from that of “joint-types JIA”. Disturbance of the innate immune system and ultimately severe cytokine hyperactivation play a major role in the pathogenesis of systemic JIA. Currently, it is widely accepted that systemic JIA is a form of autoinflammatory disease rather than an autoimmune disease [4]. Thus, the present review begins with a clinical outline and imaging findings of joint-types JIA before proceeding to a discussion of systemic JIA. It should be noted that children with a joint-type JIA are commonly antinuclear antibody (ANA)-positive and often associated with uveitis. The risk of uveitis varies with the age at onset, disease subtype, and the presence of ANA. The pathogenic relationship between arthritis and uveitis is still unknown.

## Clinical manifestations of joint-type JIA

### The oligoarticular type

Oligoarthritis is defined as the involvement of four or fewer joints within six months of onset and it is subdivided into persistent oligoarthritis (where oligoarticular involvement persists beyond six months after onset) and extended oligoarthritis (where it progresses to involvement of more than four joints beyond six months after onset). The exclusion criteria are complicated, but in principle they consist of the exclusion of psoriatic arthritis and enthesitis-related arthritis as well as RF positivity. Oligoarthritis is the most common type of JIA, accounting for about 40% of pediatric JIA cases. The peak age at onset is 2–4 years. The female to male ratio is 3:1 [5]. Only a single joint is initially affected in about a half the patients. The affected joints are not painful but only swollen and stiff in about a quarter of the patients. The knee is most frequently affected, followed by the ankle. Isolated involvement of the hip is uncommon, and systemic illness is absent. Approximately 60% of affected children are ANA-positive [5]. They have a risk of chronic uveitis, and therefore require regular ophthalmological examination. Uveitis in JIA, a common cause of blindness in children, may remain asymptomatic until the late stage when visual loss occurs. Otherwise, the prognosis is generally favorable, and most patients achieve prolonged remission. The outcome is better in persistent oligoarthritis than in the extended type, which may overlap with the RF-negative polyarticular type.

### The RF-negative polyarticular type

RF-negative polyarthritis is defined by the involvement of five or more joints within six months of onset. Affected children may show systemic symptoms, such as a mild fever and swollen lymph nodes, although these are milder than in systemic JIA. Some 40–50% of affected children are ANA-positive and have an increased risk of uveitis. Interestingly, the peak age at onset is bimodal (2–4 years and 10–14 years). The female-to-male ratio is 3:1 in the early onset group and 10:1 in the late onset group [5]. It is unclear whether or not the pathogenesis, clinical presentation, and outcome differ between the groups. However, it is tempting to assume that the former overlaps with extended oligoarthritis, while the latter overlaps with RF-positive polyarthritis.

### The RF-positive polyarticular type

RF-positive polyarthritis may be described as juvenile-onset adult RA. Its clinical features resemble those of adult RA. Adolescent females are the most commonly affected. The female-to-male ratio is 9:1 [5]. Patients may present mild systemic symptoms. As is seen in adult RA, symmetrical involvement of the small joints and carpo-tarsal joints is typical. The large joints are variably affected, but symmetrical presentation is the rule. Cervical spinal lesions are common, and atlantoaxial instability may cause serious physical disabilities. Chronic temporomandibular arthritis may cause micrognathia, and masticatory dysfunction can threaten the patient’s quality of life. The prognosis is guarded; patients tend to experience a prolonged active course and frequently experience joint destruction within five years. Uveitis and ANA positivity are uncommon.

### Psoriatic arthritis

The diagnosis of psoriatic arthritis is straightforward when psoriasis and arthritis are concurrent. However, joint involvement occasionally precedes the development of skin lesions, for which the diagnostic criteria include at least dactylitis, nail pitting and/or onycholysis, and psoriasis in first-degree relatives, in addition to arthritis. The prevalence of psoriatic arthritis is a little higher in females than in males. The onset is bimodal (2–4 years and age 9–11 years) [5]. The former age group is occasionally associated with uveitis and ANA positivity, while the latter age group may present concurrent axial lesions and HLA B-27 positivity [5, 6].

### Enthesitis-related arthritis

Enthesitis-related arthritis is the only subtype of JIA that is more common in males. The female-to-male ratio is 1:7. The peak age at onset is 9–12 years [5]. Its early manifestation is asymmetric involvement of the large joints. A unique, musculoskeletal manifestation that distinguishes this disease from the other types of JIA is lower limb enthesitis, which commonly occurs in the inferior patellar ligament, Achilles tendon, and plantar aponeurosis. Tarsitis is common, but the wrist and fingers are rarely affected. Enthesitis-related arthritis is strongly associated with HLA B-27 and shares pathogenic and clinical features with “adult” ankylosing spondylitis. Development of axial lesions (spondyloarthritis and sacroiliitis) in enthesitis-related arthritis is preceded by peripheral joint involvement. Careful monitoring of the axial skeleton, particularly for sacroiliitis, is important for a prompt and definitive diagnosis and early treatment for inflammatory back pain. Enthesitis-related arthritis is uniquely associated with acute, but not chronic, uveitis. Fortunately, the prognosis is good.

Ethesitis-related arthritis overlaps with juvenile spondyloarthropathy (JSpA), a diagnostic label for children with a combination of arthritis, enthesitis, and axial lesions, including not only enthesitis-related arthritis and psoriatic arthritis, but also reactive arthritis (formerly known Reiter’s syndrome) and arthropathies associated with inflammatory bowel disease (IBD). The latter two are not included in the ILAR classification. However, IBD is frequently associated with enthesitis-related arthritis, suggesting a pathogenic relationship.

## Imaging diagnosis of joint-type JIA

Imaging examinations contribute to the diagnosis and management of JIA and are utilized to determine the presence and activity of synovial inflammation, monitor disease progression, and exclude other joint diseases. Imaging protocols for JIA have not been firmly established compared with those for adult rheumatoid arthritis. Optimal imaging studies should be individualized.

**Fig. 1:**
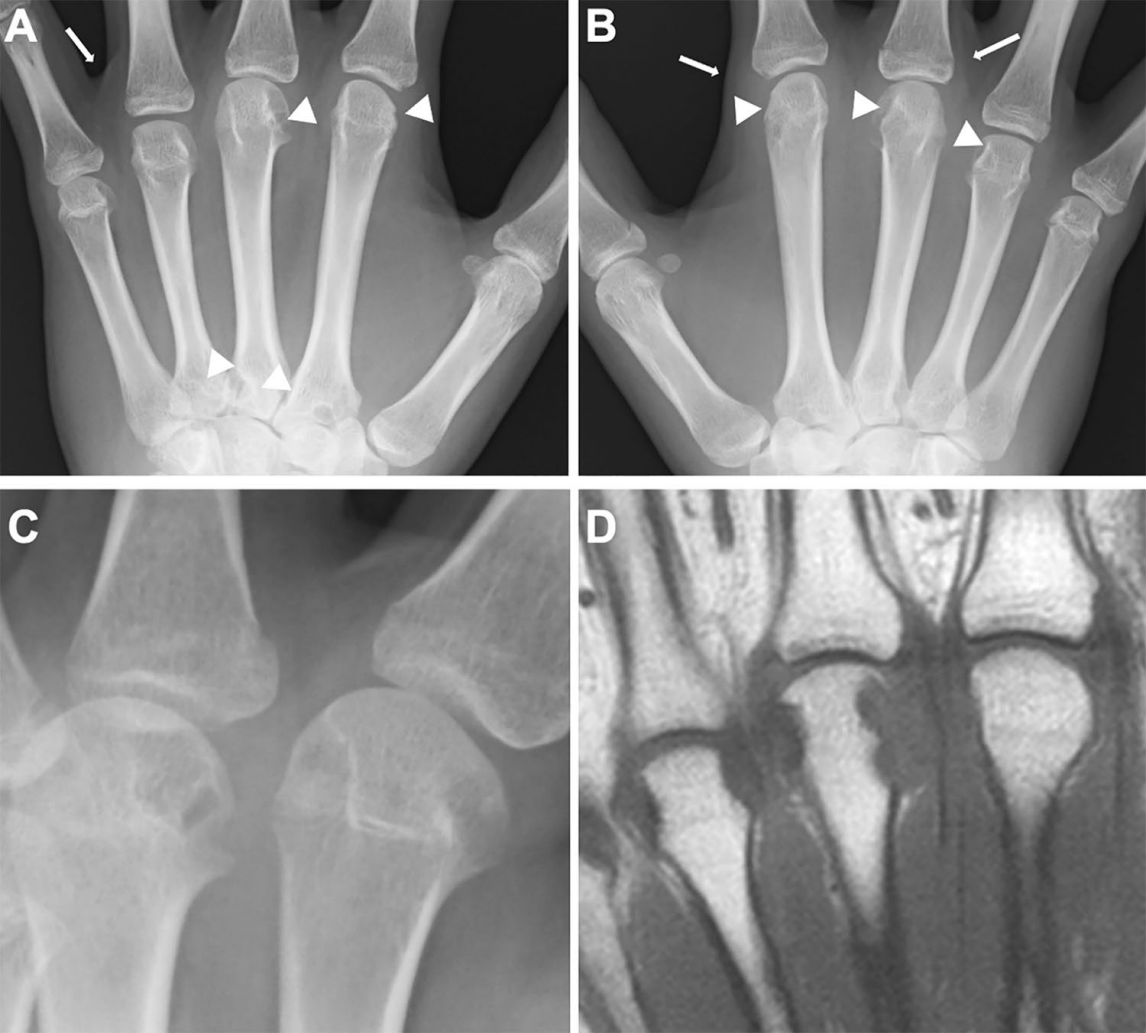
RF positive polyarticular type JIA. **A–D**. A 14-year-old girl with painful hands. The frontal radiographs of the hands showed marginal erosions of the distal end of the left 2nd and 3rd metacarpals (arrowheads, A), the proximal end of the left 3rd and 4th metacarpals, and the distal end of the right 2nd, 3rd, and 4th metacarpals (arrowheads, **B**). Periarticular soft tissue swelling was also discernible (arrows, **A**, **B**). The close-up view of the left hand (**C**) and T1-weighted coronal MRI image of the left hand (**D**) delineated more clearly marginal erosion of the metacarpals

**Fig. 2:**
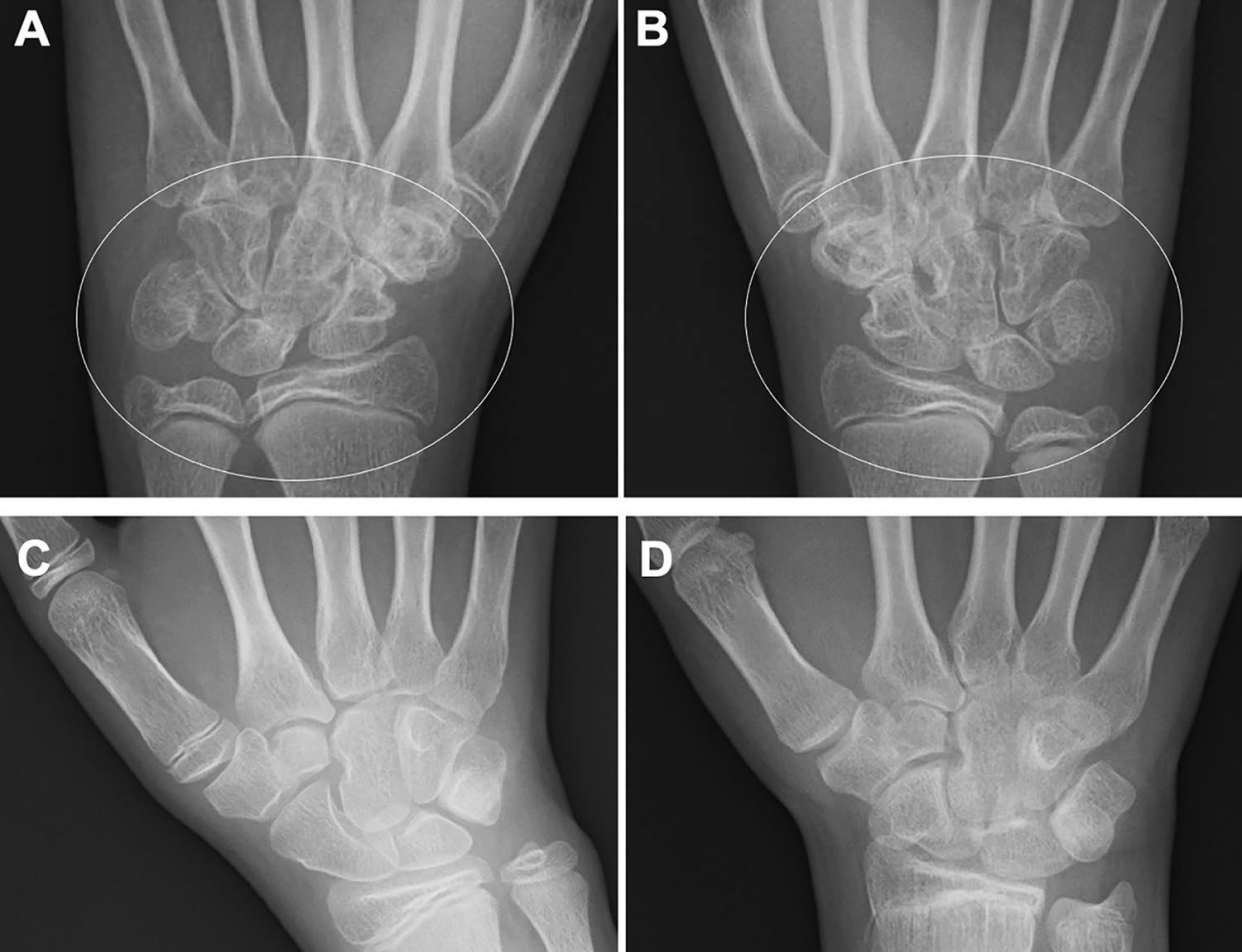
Two cases with RF positive polyarticular type JIA. **A–B**. A 14-year-old girl. The frontal radiographs of the hands showed bilateral, symmetric carpal erosions and carpal joint space narrowing (circles, **A**, **B**). **C–D**. A different girl. The radiographs showed progression of joint space narrowing and development of carpal erosion between age 8 years (**C**) and 12 years (**D**)

**Fig. 3:**
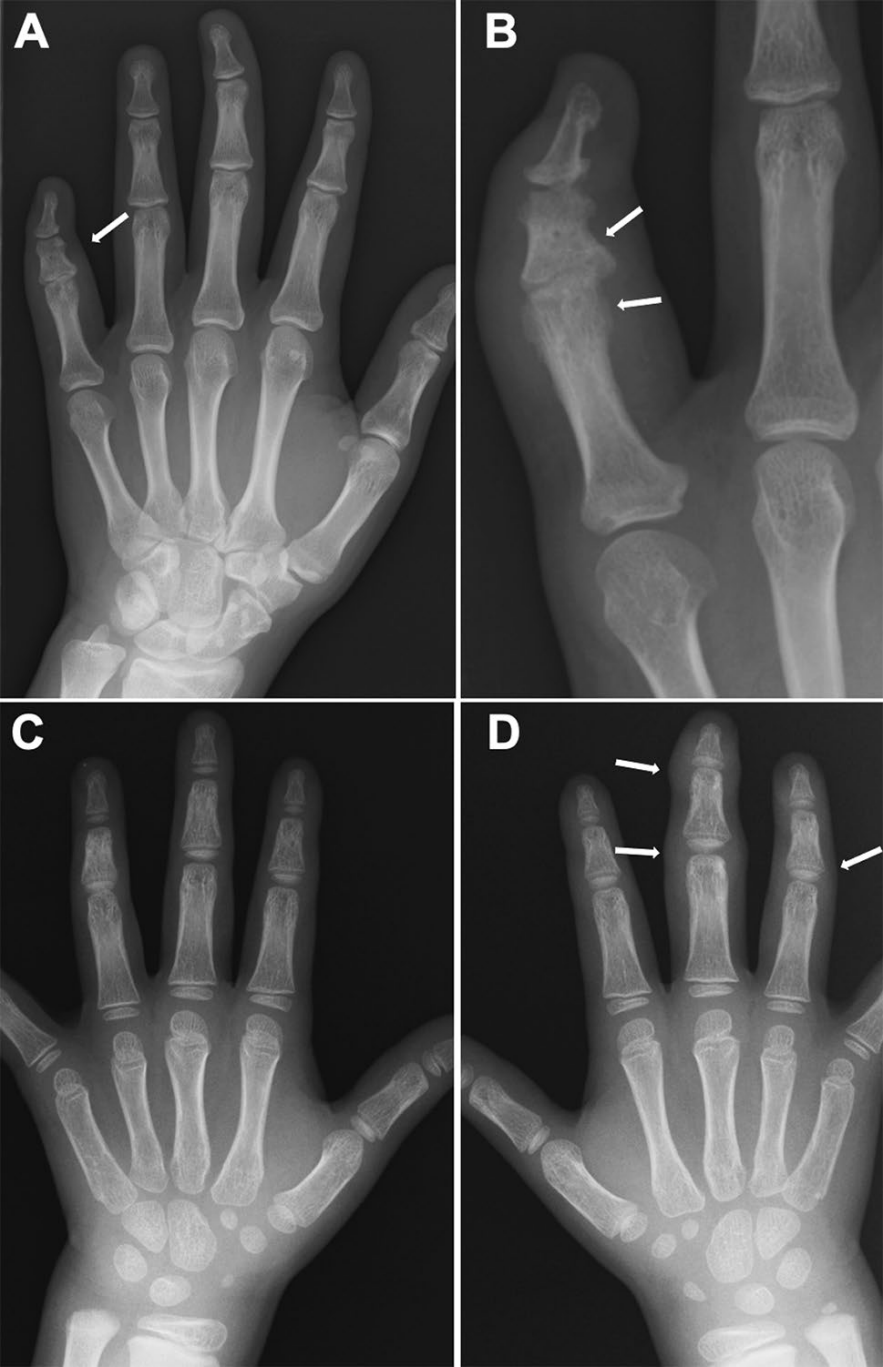
Two cases with sausage fingers with or without dactylitis. **A–B**. A 15-year-old girl with psoriatic arthritis, who initially presented with swelling of the left 5th finger and developed psoriasis 2 years later. The frontal radiographs of the hands showed sausage-like soft tissue swelling, joint space narrowing of the PIP, and dactylitis of the middle phalanx of the left fifth finger (arrow, **A**): dactylitis was depicted as moth-eaten type-trabecular derangement (arrows, **B**). **C-D**. A 5-year-old girl with oligoarticular JIA. The frontal radiographs of the hands showed sausage-like swelling of the right 3rd and 4th fingers (arrows, **D**); carpal ossification of the right wrist is accelerated as compared with that of the left (**C**). Courtesy of Professor T Hasegawa, Department of Pediatrics, Keio University School of Medicine

**Fig. 4:**
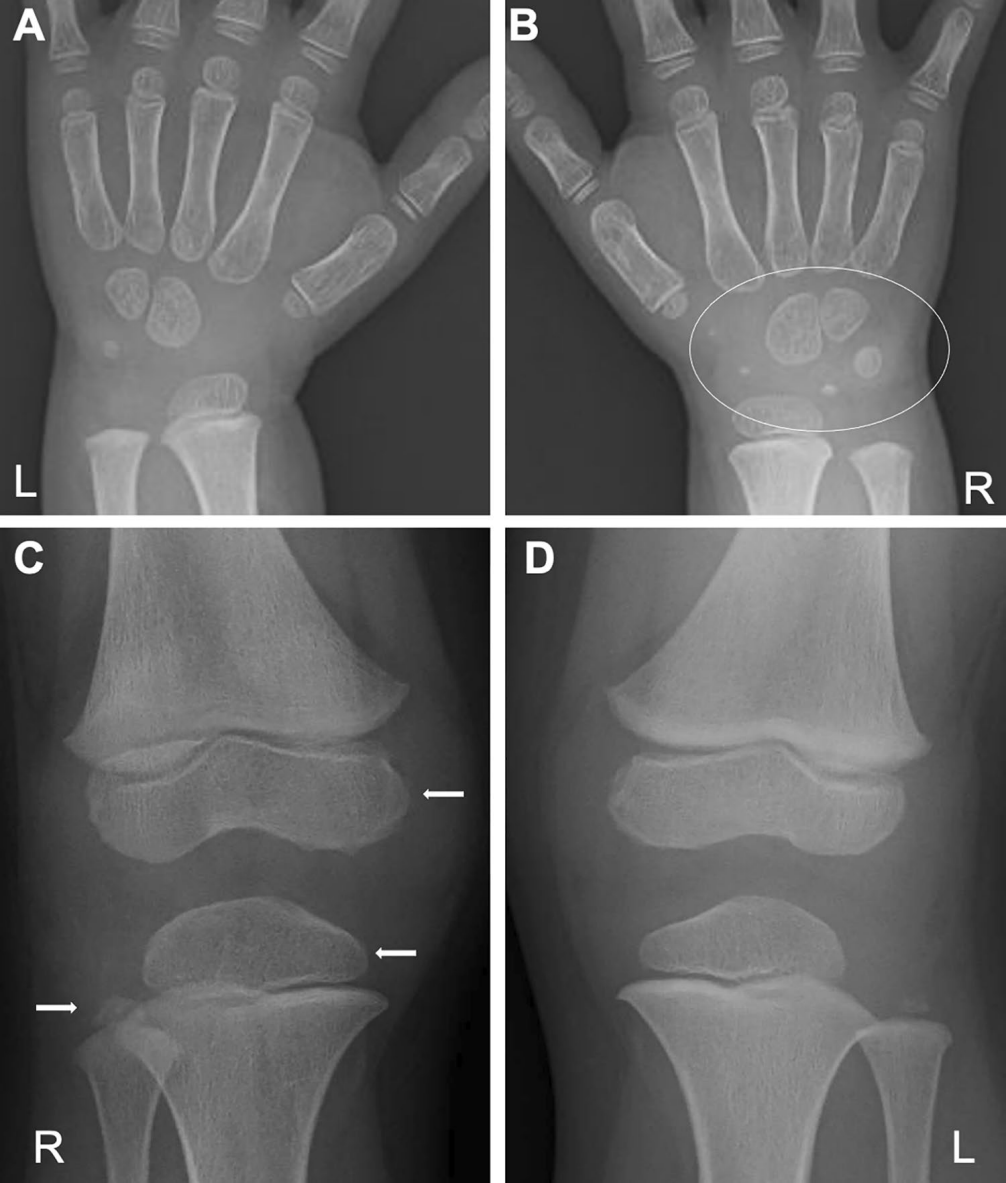
Two cases with oligoarticular type JIA. **A-B**. A 2-year-old girl with restricted dorsiflexion of the right wrist. The frontal radiographs showed accelerated carpal ossification of the right (circle, B) and a normal left hand (**A**). **C–D**. A 3-year-old boy with painful right knee. The frontal radiographs showed large epiphyses (arrows, **C**) and juxtaarticular osteopenia of the right knee and a normal left knee (**D**)

Sequelae common to both joint-types JIA and adult RA include 1) acute synovitis with increased joint fluid, periarticular soft tissue edema, and periarticular osteopenia (Fig. 1A, B), 2) chronicity of synovitis with pannus formation (Fig. 1C, D), 3) pannus augmentation with centripetal erosion of the cartilage and subchondral bone (beginning with marginal erosion at the synovial attachment and progressing centrally) (Fig. 2A, B), and 4) simultaneous occurrence of chondrolysis causing joint space narrowing and further joint destruction (Figs. 1, 2) (Fig. 2C, D).Inflammatory synovitis commonly occurs in the tendon sheaths of the hands and feet, causing sausage fingers (toes) and dactylitis (Fig. 3). Tenosynovial inflammation at the tendon attachment results in enthesitis. Dactylitis and enthesitis are common in psoriatic arthritis and enthesitis-related arthritis.

**Fig. 5:**
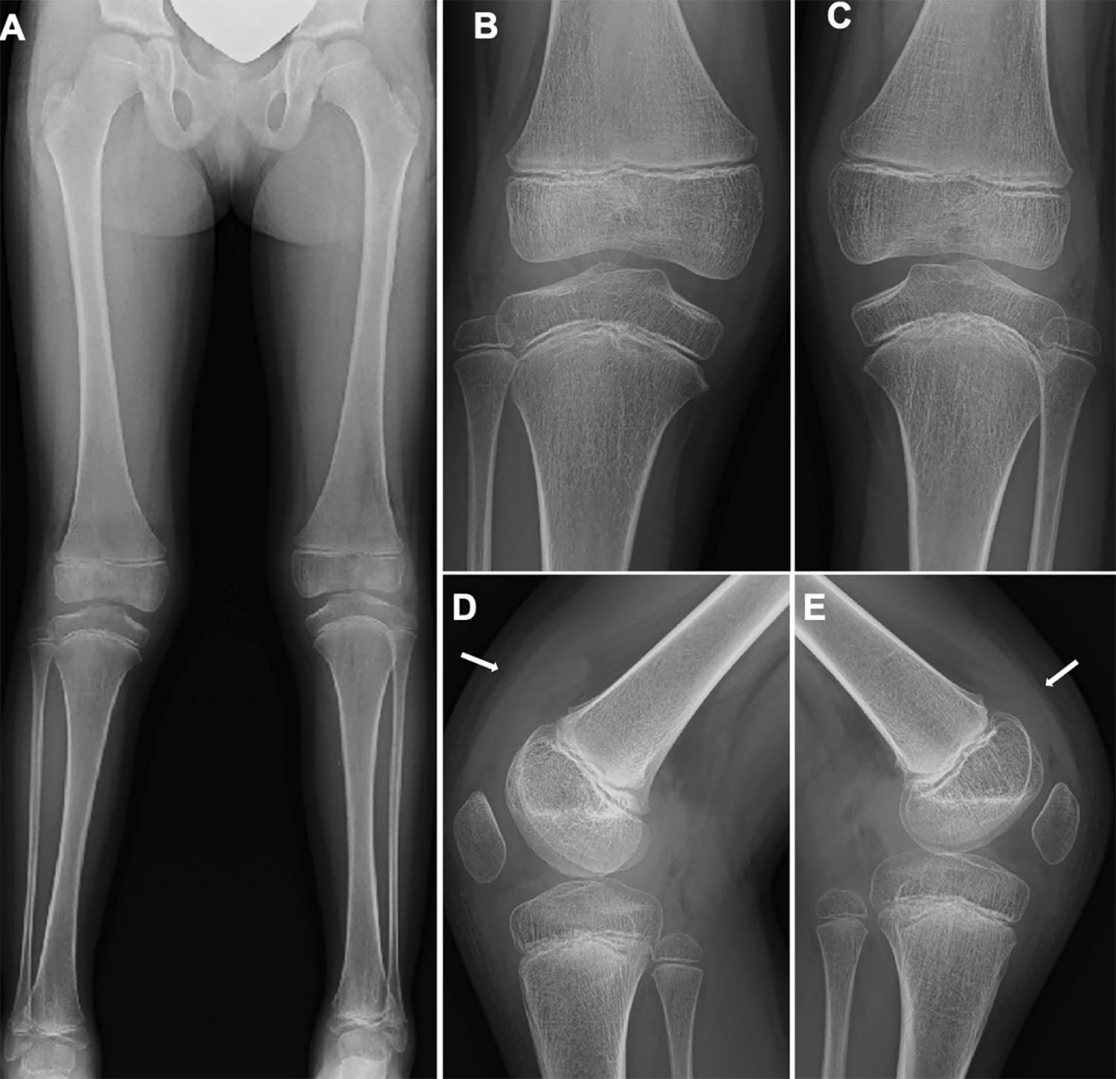
Oligoarticular type JIA. **A–E**. A 6-year-old girl with intermittent pain of bilateral knee joints that started at age 2 years, but remained undiagnosed until age 6 years. The frontal radiograph of the lower extremities showed leg length discrepancy with right genu valgum (**A**). The frontal radiograph of the knees showed large, squared epiphyses and the metaphyseal broadening of both knees. The tibial growth plates show upward convexity instead of a normal horizontal direction (**B**, **C**). The lateral radiograph of the knees depicted dilatation of the suprapatellar bursae of the right knee (arrow, D) and to a lesser extent of the left (arrow, **E**)

Moreover, there are imaging findings specific to JIA. Chronic synovitis modifies endochondral bone growth by accelerating epiphyseal ossification (Fig. 4A, B), enhancing physeal endochondral ossification (Figs. 4C, D, 5), and promoting premature fusion of the growth plate [7] (Figs. 4, 5). These findings often provide clues for diagnosing JIA particularly in young children, in whom bone erosion develops at a relatively later stage owing to the thicker epiphyseal cartilage.

### Radiography

**Fig. 6:**
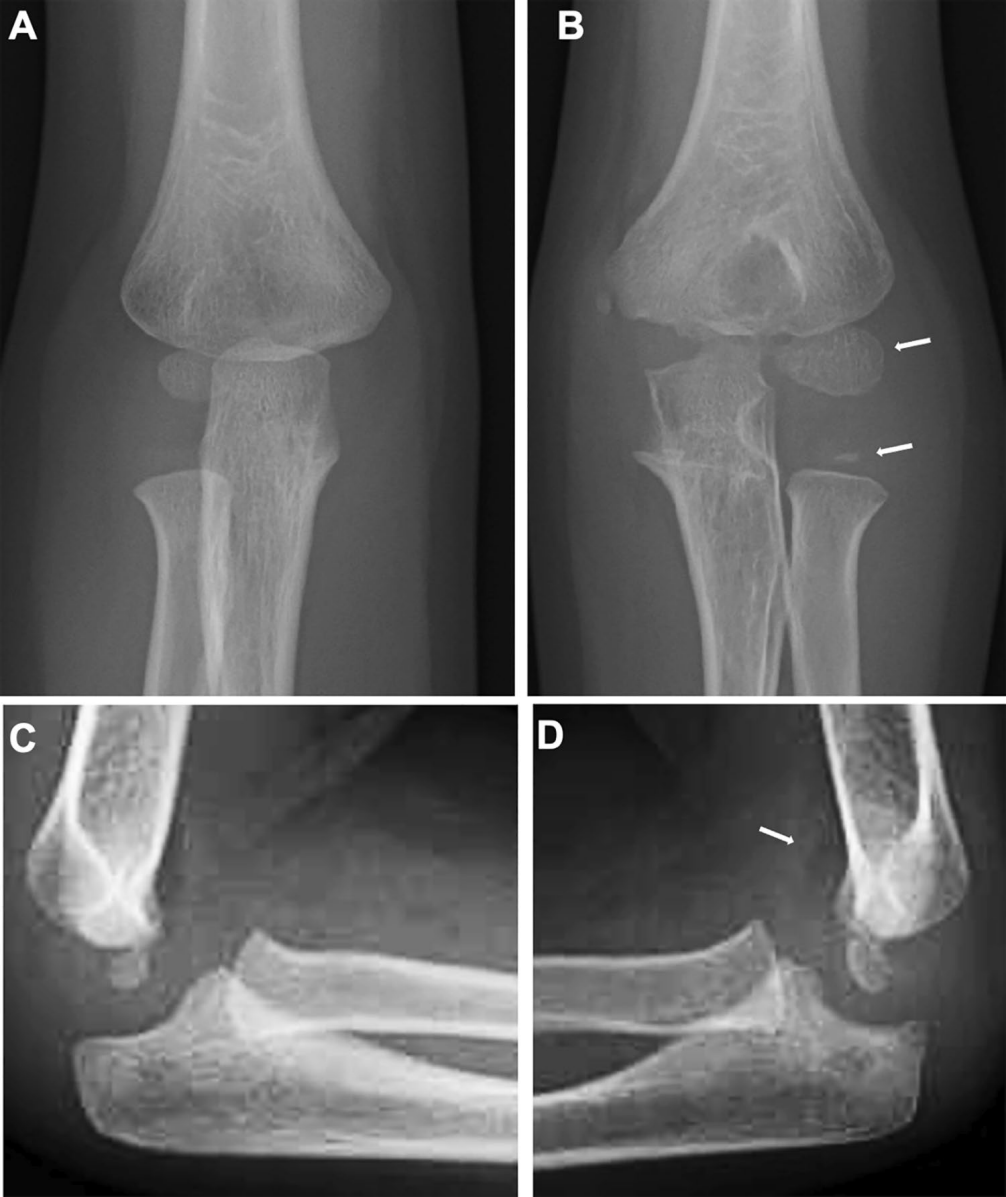
Oligoarticular type JIA. **A–D**. A 3-year-old girl with limited extension of the left elbow. The right elbow was normal (**A**, **C**). The left elbow showed accelerated epiphyseal ossification (arrows, **B**), metaphyseal broadening, and joint distension (**B**, **D**). The joint distension was depicted as mildly elevated anterior fat pad (arrow, **D**)

Conventional radiography is the primary imaging tool for evaluating musculoskeletal abnormalities. However, radiography cannot directly delineate synovial proliferation or assess for the presence of active inflammation. In the early stage of JIA, radiography can exclude non-rheumatic disorders with painful joints, such as traumatic lesions, neoplasms, and bone infections, and also can detect indirect signs of synovitis, such as joint distension, juxta-articular soft tissue swelling, and periarticular osteopenia [7, 8]. Joint distension of the knee, elbow, and ankle is discernible as an enlargement of the suprapatellar bursa, elbow fat pad sign, and ankle tear drop sign, respectively (Figs. 5, 6). Joint distension of the hip, shoulder, and wrist is more difficult to ascertain.

As the disease stage of JIA progresses, radiological manifestations of abnormal endochondral development and impairment of normal modeling provide clues to the diagnosis of JIA. These include epiphyseal enlargement and squaring, increased transverse diameter of the growth plate, metaphyseal broadening, accelerated carpal ossification, and overgrowth of the extremities (Figs. 4, 5, 6) [8] (Figs. 4, 5, 6). This early bone overgrowth culminates in growth retardation. Marginal, bony erosions, joint space narrowing, and eventually joint deformity or malalignment are late sequelae (Figs. 1, 2). Enthesitis appears as an irregularity/spur at the insertion of tendons, ligaments, and fascia. Dactylitis initially presents as periosteal new bone formation along with sausage-like swelling of the digits and later evolves into trabecular abnormalities with a moth-eaten appearance (Fig. 3).

### Ultrasound (US)

**Fig. 7:**
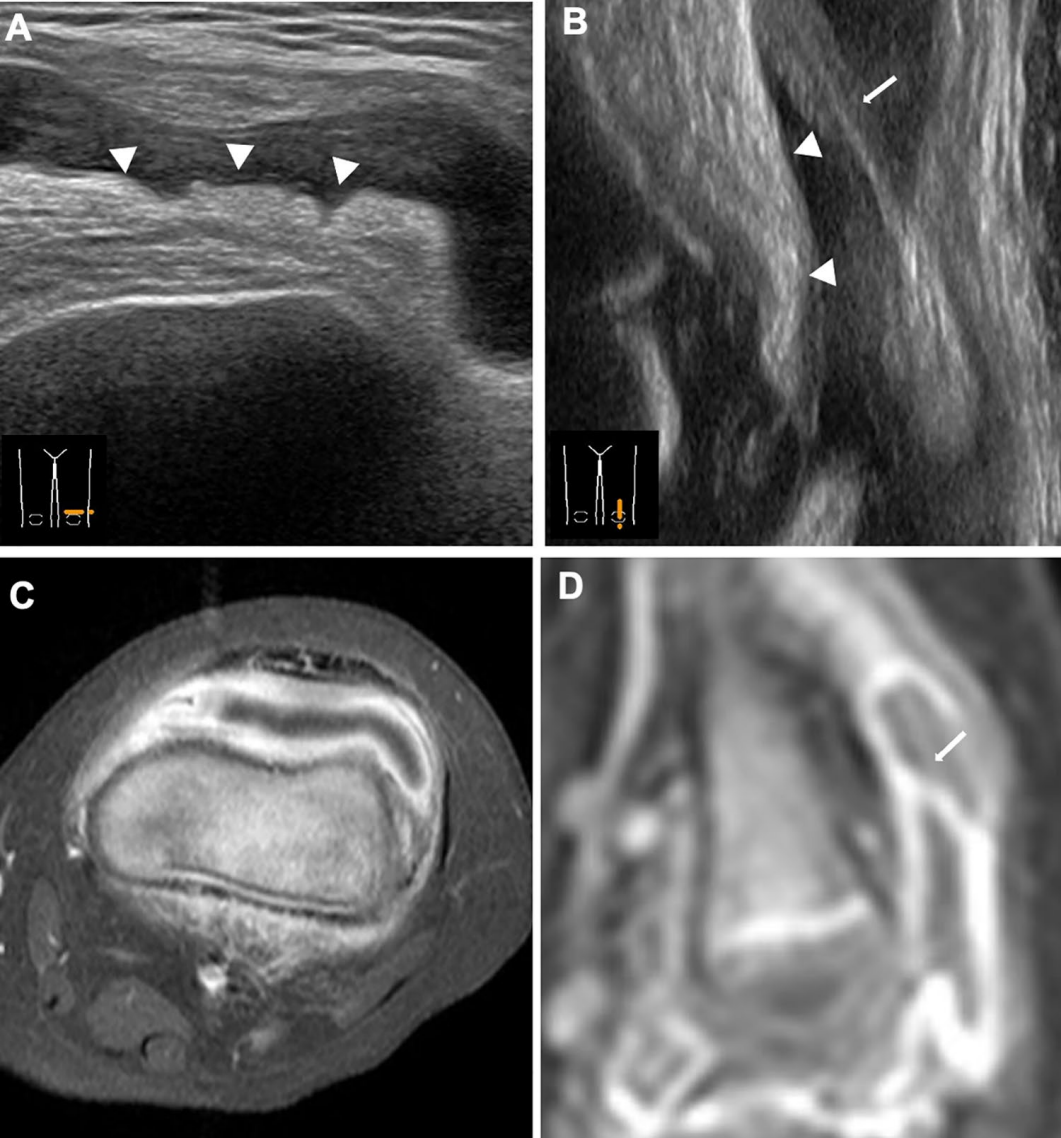
US images for synovitis. **A–D**. An 18-month-old boy with painful left knee joint. Transverse (**A**) and longitudinal (**B**) view of US depicted a heterogeneously thickened, partially wavy synovium (arrowheads, **A**, **B**) and increased joint fluid, corresponding to those of post-contrast enhanced axial MRI (**C**) and the late phase of dynamic contrast-enhanced sagittal MRI (**D**), respectively. Note thickening of the suprapatellar plica due to its synovial thickening (arrows, **B**, **D**)

US has gained in popularity as an imaging tool for the initial diagnosis of JIA and follow-up examinations. Its advantages include no need for sedation and inexpensiveness. A high-frequency US probe (8–15 MHz) is suitable for musculoskeletal assessment. US can easily be used to evaluate joint fluid and synovial proliferation (Fig. 7). Color Doppler imaging can also display hyperemia of the affected joints and neighboring soft tissue, which is particularly useful when evaluating small joints. Unlike radiography, US directly displays abnormalities of tendons, ligaments, and even cartilage and bone at the synovial attachment, although it is unable for assessing for bone marrow changes. A thickened synovium appears as a hypoechoic, compression-deformable structure within the joint capsule. US can identify joint fluid, even if it is physiological and present in only small amounts [2, 9]. Tenosynovitis appears as thickening of the tendon sheath associated with irregular hypoechoic areas. The normal articular cartilage appears as uniformly hypoechoic, smooth structures on the bony surface. As the disease progresses, the cartilage becomes heterogeneously thickened, then thinned, and finally disappears completely, leading to bone erosion.

### MRI

**Fig. 8:**
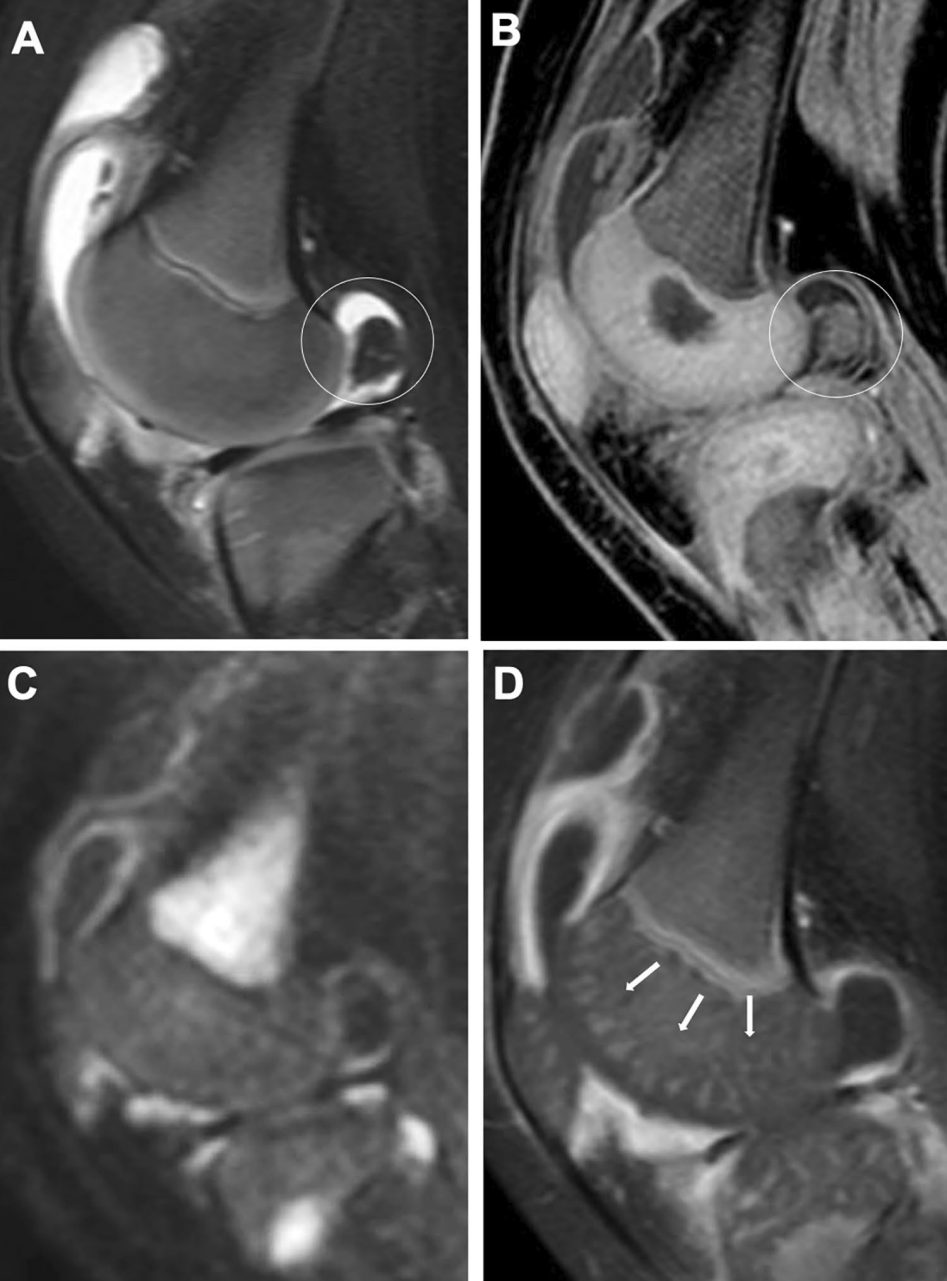
MRI for synovitis. **A–D**. A 1-year-old infant with painful left knee. Fat-suppressed T2-weighted (**A**) and water selective gradient echo sagittal images (**B**) showed a large pannus in the posterior fossa (circles, **A**, **B**). The articular cartilage was differentiated from the epiphyseal cartilage on both sequences. Diffusion-weighted (**C**) and Gd-enhanced T1-weighted sagittal images (**D**) showed high intensities of the synovium, indicative of active synovitis, whereas the popliteal pannus appears black and indistinguishable from joint fluid on these sequences. Vascular structures in the epiphyseal cartilage were well seen on Gd-enhanced image (arrows, **D**)

**Fig. 9:**
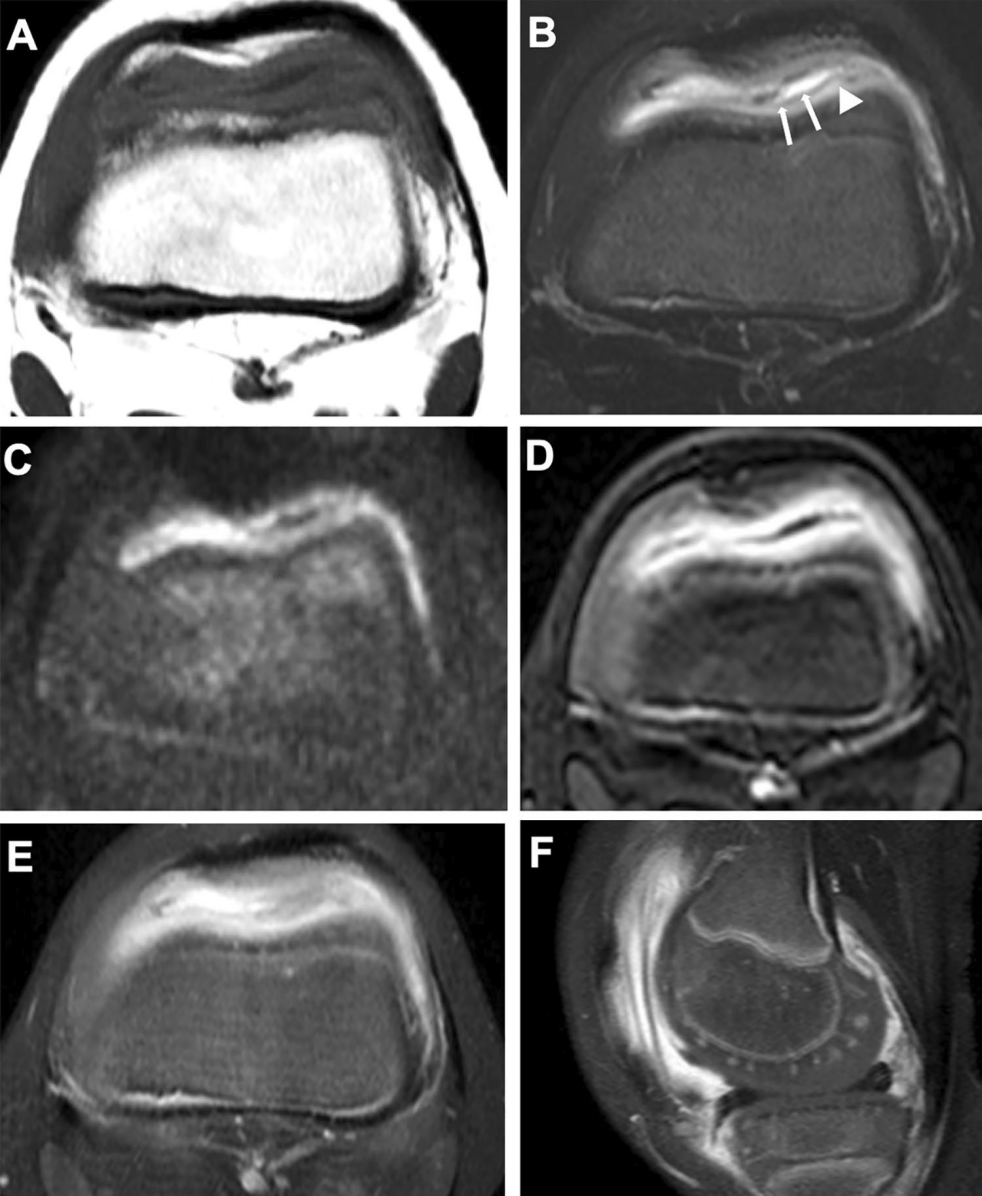
MRI in oligoarticular type JIA. **A–F**. A 4-year-old girl with painful left knee. T1-weighted image (**A**) and fat-suppressed T2-weighted images (**B**) of the left knee demonstrated thick synovial tissues. T2-weighted image reveals low signal of the superficial layer (arrows) and intermediate signal of the deep layer (arrowhead) of the thick synovium, contrasting with high signals of joint fluid (**B**). Axial DWI showed high-intensity synovium and low intensity joint fluid (**C**). Dynamic contrast enhanced axial image at the early phase showed prominent synovial enhancement (**D**). Contrast-enhanced fat-suppressed axial (**E**) and sagittal (**F**) T1-weighted image showed diffusion of contrast materials into the joint cavity and hyperemia (prominent vascular structures) in the epiphyseal cartilage

**Fig. 10:**
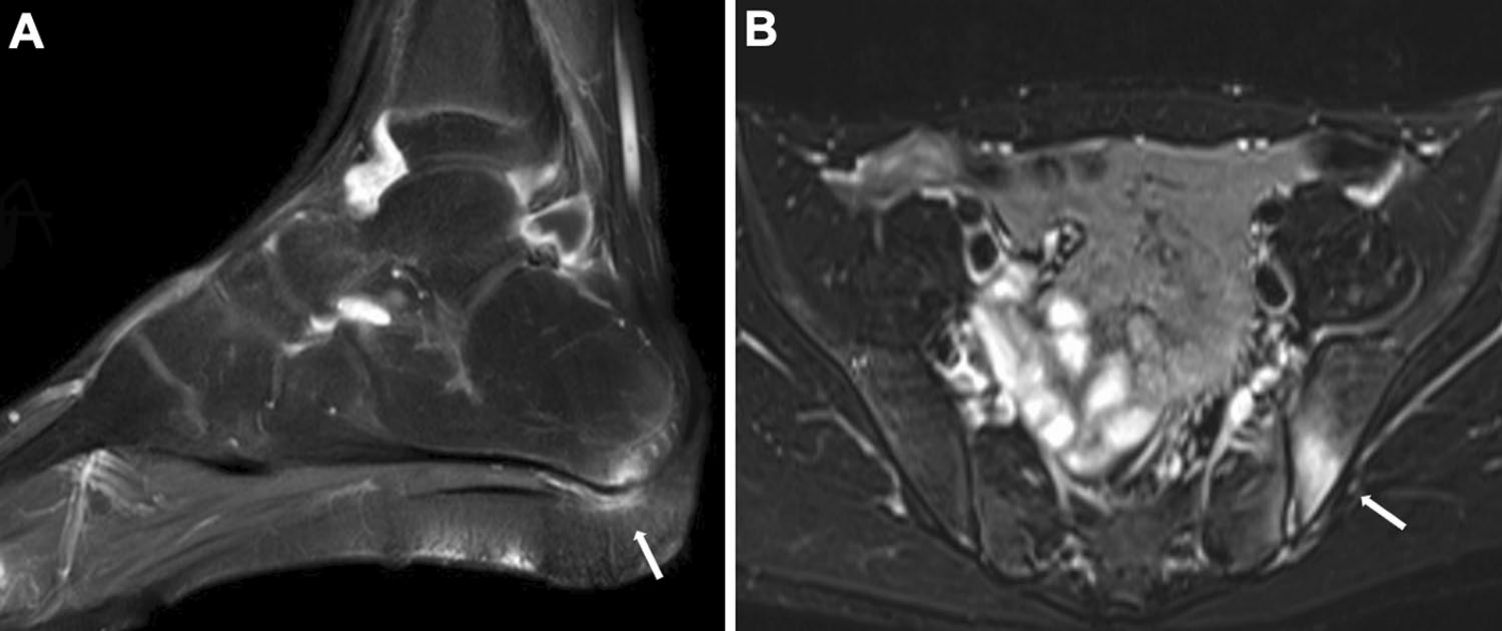
Enthesitis-related arthritis: **A–B**. A 12-year-old boy with painful left foot and positive HLA B-27. Fat-suppressed-T2-weighted sagittal image showed high signal intensities at the attachment of the plantar fascia to the calcaneus (arrow, **A**). Three years later, he developed left sacroiliitis that was asymptomatic (arrow, **B** fat-suppressed-T2-weighted axial image)

**Fig. 11:**
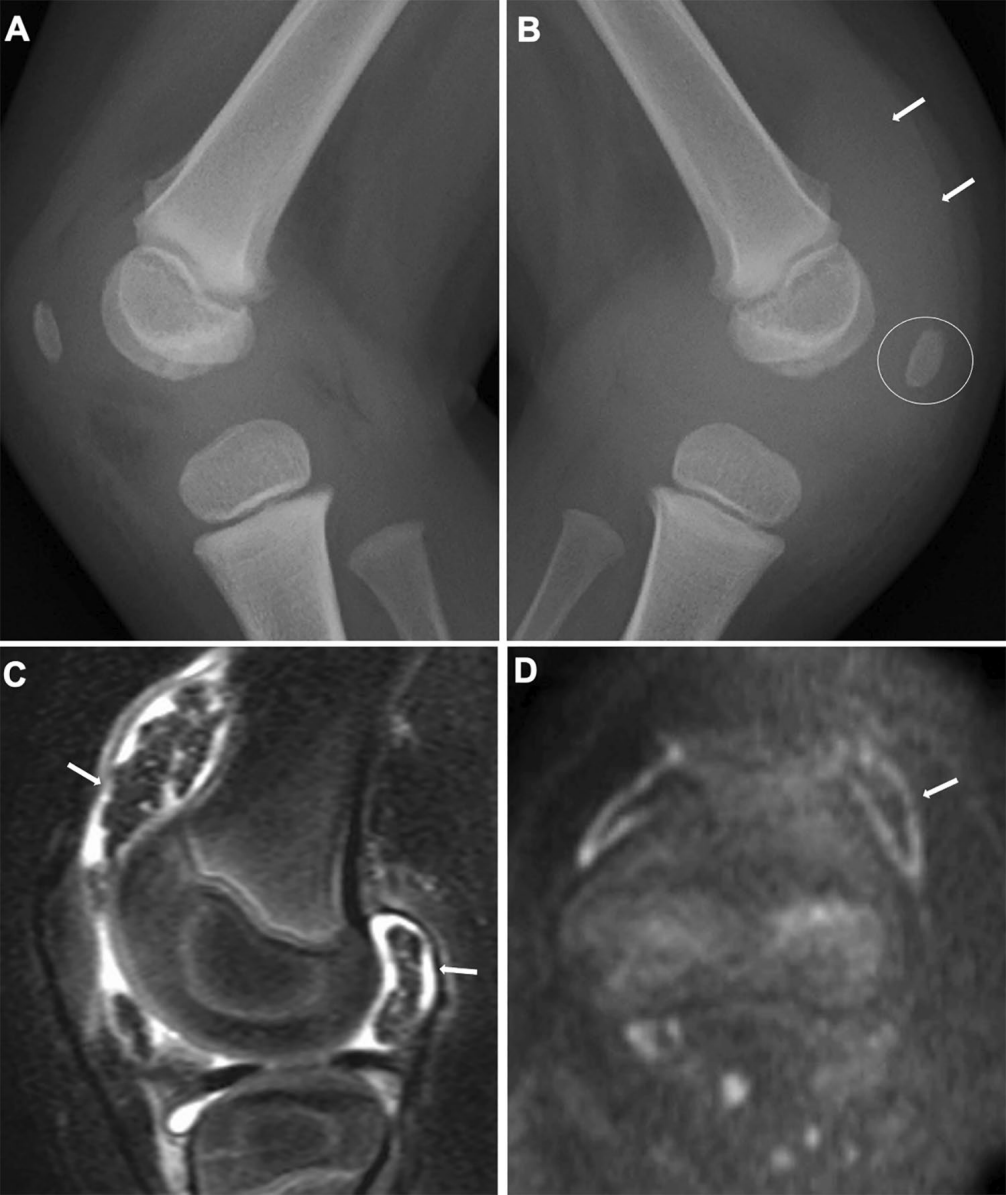
Oligoarticular type JIA. **A-D**. A 5-year-old girl with left knee pain. The lateral radiographs of the knees showed distension of the suprapatellar bursa (arrows, **B**) and accelerated patellar ossification (circle, **B**) of the left knee and a normal finding of the right knee (**A**). In the left knee, sagittal fat-suppressed T2-weighted sagittal image showed low signal rice bodies (arrows, **C**), and axial DWI demonstrated high signal intensities of the synovium, suggesting active synovitis (arrow, **D**)

MRI is the optimal tool for assessing joint changes in JIA and can demonstrate the normal aspects and abnormalities in the synovium, joint capsules, ligaments, tendons, cartilage, and subchondral bone. The disadvantages of MRI are the need for sedation in young patients, the expense involved, and the long time required to perform it. MR sequences optimal for JIA have not yet been standardized. Fluid-sensitive imaging (typically fat-suppressed T2-weighted imaging) and contrast-enhanced fat-suppressed T1-weighted imaging are commonly used to assess morphological details (synovium, periarticular soft tissues or bone). Gradient echo imaging is currently used for cartilage assessment, for which a number of new sequences have been proposed. Recently, diffusion-weighted imaging (DWI) has come to be used widely as well. Different signal properties of the articular and juxta-articular tissues on different image sequences can be seen in the same patient (Fig. 8). Contrast-enhanced MRI enables assessment of the severity of hyperemia and increases in blood flow, by which in turn inflammation activity can be assessed. In addition, epiphyseal cartilage may demonstrate a spoke-wheel-like contrast effect, reflecting intracartilaginous hyperemia or increased vasculogenesis [8] (Fig. 9). MRI is the only modality that can assess bone marrow signal abnormalities and the composition of articular cartilage [10] and also plays a cardinal role in the evaluation of inflammatory changes in the axial skeleton (e.g., apophyseal joints of the spine and sacroiliac joints), temporomandibular joints, and subtalar joints, all of which are difficult to evaluate with plain radiography or US [8, 11] (Figs. 10, 11, 8).

#### Synovium

The normal synovium has such thin and low signal intensity that it cannot be differentiated from the joint capsule on water-sensitive imaging. On the other hand, the inflamed, thickened synovium (synovitis) appears as a linear, wavy or nodular capsular lining with mildly low to intermediate signal intensity contrasting with the very high signal intensity of joint fluid (Figs. 8, 9). Intravenous administration of Gd-based contrast agents allows more detailed evaluation of the synovium. The normal synovium presents as a uniformly thin lining with modest contrast enhancement, while synovitis appears as a lumpy lining with heavy contrast enhancement (Fig. 9) (Figs. 8, 9).

Dynamic contrast-enhanced MRI is useful for determining inflammation activity. Contrast enhancement of active synovitis is most conspicuous in the early phase (one to three minutes after contrast injection). Diffusion of the contrast agent from the synovium into the joint cavity in the late phase hampers image interpretation (Fig. 9). Recent reports have indicated that DWI may distinguish active synovial inflammation from inactive one [12, 13] (Fig. 11) (Figs.8, 9, 11). Chronic, fibrous panni demonstrate areas of low intensity on T2-weighted imaging. Detached panni or rice bodies demonstrate areas of low intensity as intra-articular free bodies resembling those of synovial osteochodromatosis (Fig. 11).

#### Tenosynovitis and enthesitis

On fluid-sensitive imaging, tenosynovitis appears as thickening of the tendon with increased intrinsic signals and fluid collection in the tendon sheath. Likewise, enthesitis manifests thickening of the tendon or ligament and bone marrow edema at the tendinous or ligamentous attachment. In advanced cases, osteophyte formation and cortical irregularities may be seen. The presence of the enthesitis has a high index of suspicion of psoriatic arthritis and enthesitis-related arthritis (Fig. 10).

#### Bone and cartilage

**Fig. 12:**
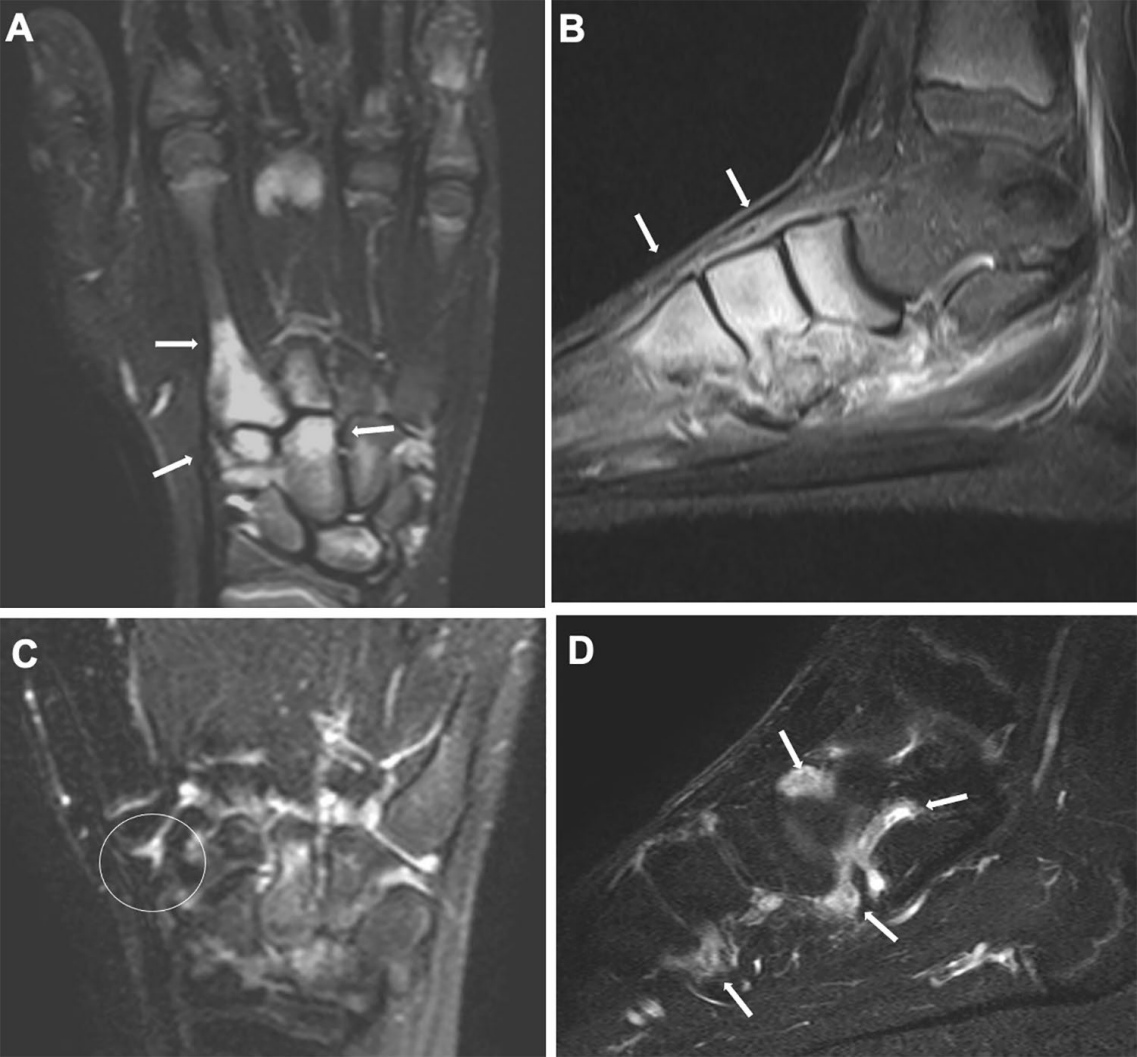
RF-positive polyarticular type JIA. **A-D**. An 8-year-old boy. Fat-suppressed T2-weighted coronal image of the hand (**A**) and sagittal image of the foot (**B**) showed extensive bone marrow edema of the carpals, metacarpals, tarsals, and metatarsals (arrows, **A**, **B**). After 6 months of treatment, bone marrow edema had reduced. However, carpal erosions (circle, **C**) and diffuse synovial thickening of the intertarsal joints (arrows, **D**) became manifest

Most conventional sequences (T1-weighted imaging, T2-weighted imaging, proton-density-weighted imaging, and gradient-echo imaging) clearly distinguish the articular cartilage from the subchondral bone and joint fluid and demonstrate cartilage irregularities and thinning. Recent types of cartilage-sensitive imaging can visualize the normal zonal architecture of the articular cartilage. Fluid-sensitive sequences can reveal subchondral bone marrow edema. It has been shown that abnormal bone marrow signals correlate with the risk of the development of bone erosion and joint dysfunction in adult RA, because the signal changes stem not only from edema but also from inflammatory cell infiltration, a precursor of bone destruction [14–16] (Fig. 12). However, we should be aware of T2-high speckling in normal bone marrow in children, which is particularly common in the ankle. There may be well-demarcated signal changes in the carpal bone, referred as to carpal depression.

### The cervical spine

**Fig. 13:**
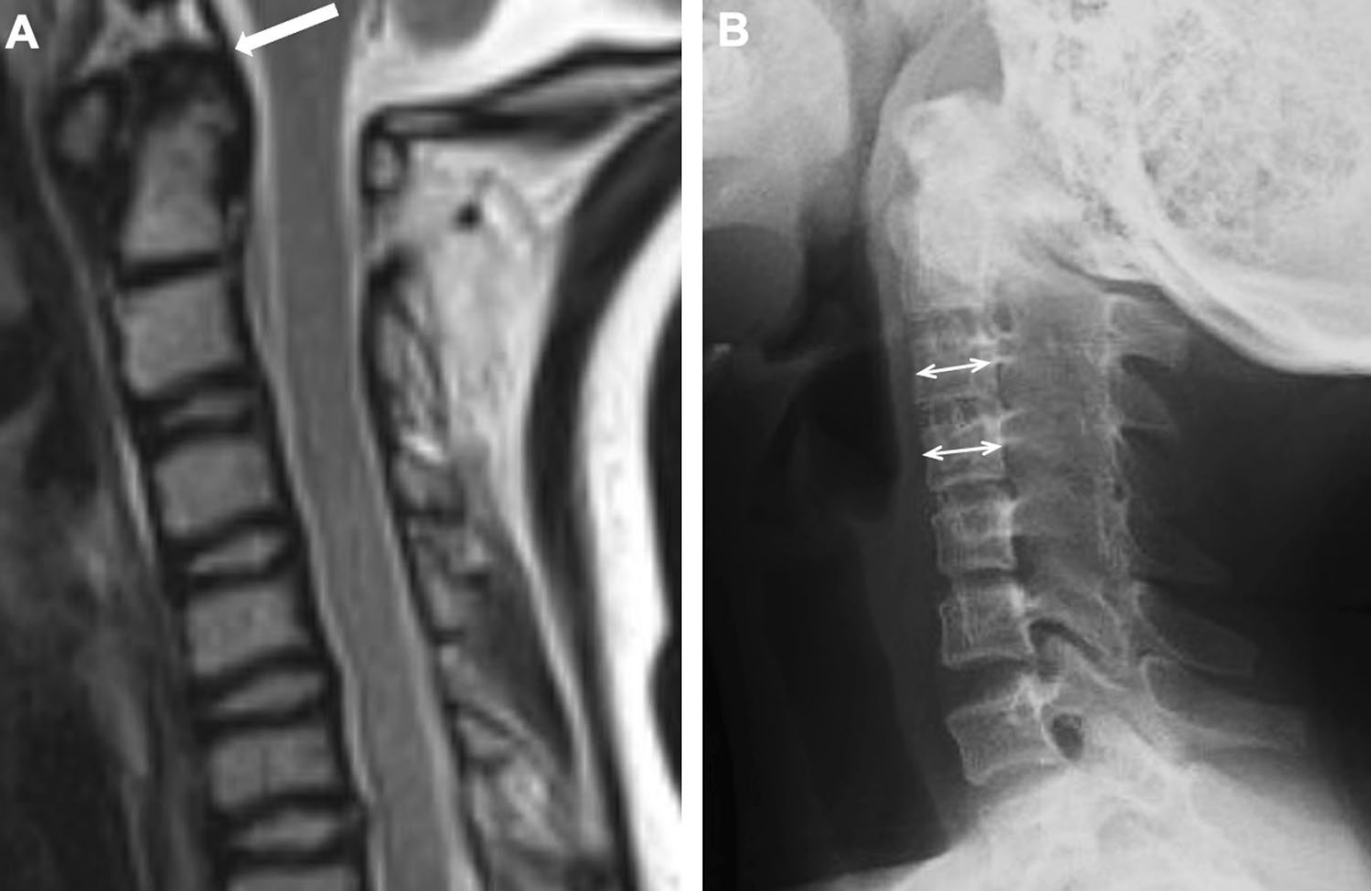
Cervical spine involvement in JIA. **A**. A 6-year-old girl with oligoarticular type JIA. Synovial hyperplasia of the atlantoaxial joint was depicted as low signal soft tissue prominence of the juxtaodontal region on T2-weighted sagittal image (arrow).** B**. A 12-year-old girl with systemic JIA. The lateral radiograph of the cervical spine showed ankylosis of the facet joints and reduced anteroposterior diameter of the vertebral bodies (double-headed arrows)

The cervical spine is sometimes involved in pediatric JIA, particularly in RF-positive polyarthritis, and the rate of involvement may be as high as 35–60% [17, 18]. In the early stage, it tends to be asymptomatic but later progresses to rigid neck and atlantoaxial subluxation with erosion of the odontoid process. Radiographic manifestations include ankylosis of the posterior neural arches and hypoplasia of the vertebral bodies (reduced anteroposterior and transverse diameter of the vertebral bodies) (Fig. 13) [19]. MRI is beneficial for detecting early atlantoaxial changes, i.e., periodontal synovial hyperplasia and bone marrow edema. Atlantoaxial rotatory fixation, on the other hand, is a rare complication.

### The temporomandibular joint (TMJ)

TMJ complications can occur in any type of JIA and are more prevalent than generally thought. A report on TMJ screening using MRI demonstrated that more than 60% of patients had TMJ arthritis [11, 20, 21]. TMJ arthritis is often asymptomatic in the early stages. However, the late functional morbidity is serious. TMJ involvement inhibits growth of the mandibular condyle and creates micrognathia [20]. MRI demonstrates increased joint effusion, abnormal synovial contrast enhancement, condylar head erosion and/or subchondral sclerosis, and disk deformity and deviation.

## Differential diagnosis of joint type JIA

This section discusses the differential diagnosis between oligoarticular type JIA, particularly cases with monoarticular involvement and other joint diseases, including pyogenic arthritis, intraarticular osteoid osteoma, tenosynovial giant cell tumor, and hemophilic arthropathy. All these disorders, like JIA, present non-specific synovial changes (synovial thickening and abnormal contrast enhancement with increased joint effusion) and may be associated with juxta-articular soft tissue edema and bone marrow edema.

Synovitis in JIA develops insidiously; however, the actual joint swelling is often erroneously identified as acute joint swelling, which raises the suspicion of pyogenic arthritis. In general, inflammatory symptoms are more severe in pyogenic arthritis [22], but distinguishing the conditions clearly is difficult. Plain radiography is unhelpful in the acute phase. In both disorders, MRI reveals synovial changes, juxta-articular soft tissue edema, and bone marrow edema. However, the soft tissue edema and marrow edema tend to be more extensive in pyogenic arthritis. Pyogenic arthritis is mostly associated with metaphyseal osteomyelitis. Presence of an intraosseous and/or subperiosteal abscess is diagnostic of pyogenic arthritis. Diffusion-weighted imaging may be the most beneficial method of detecting an abscess in its early stage.

**Fig. 14:**
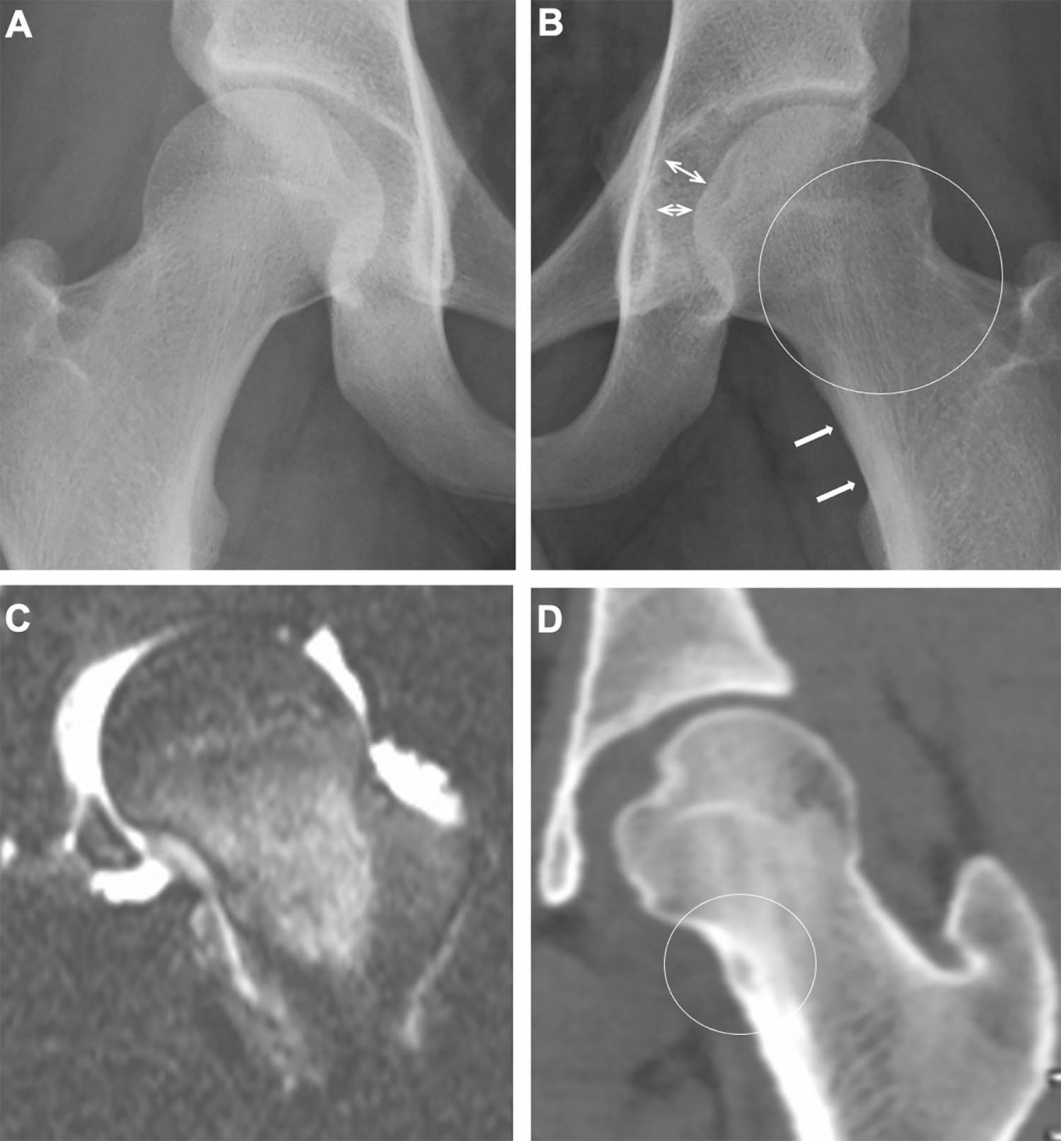
Intraarticular osteoid osteoma. **A–D**. A 17-year-old boy with painful left hip. The frontal radiographs of the hips showed mild hyperostosis of the medial cortex (arrows, **B**) and osteopenia of the left femoral neck (circle, **B**) and mildly increased acetabulo-femoral distance (double headed arrows, **B**) and a normal right hip (**A**). Fat-suppressed T2-weighted coronal image showed bone marrow edema of the femoral neck along with increased joint fluid and irregular synovial surfaces (**C**). CT revealed a small nidus in the osteosclerotic medial cortex of the femoral neck (circle, D)

Osteoid osteoma is a benign bone tumor with a predilection for the second decade of life. It is characterized by a nidus composed of osteoid tissues surrounded by reactive osteosclerosis. When arising within a joint, it causes reactive synovitis, and affected children present with a painful joint mimicking monoarticular JIA [23]. Intra-articular osteoid osteoma presents less severe osteosclerosis than cortical lesions; thus, its nidus may be barely visible on plain radiography. The signal properties of the nidus are variable on MRI, which may hamper its identification. CT is therefore the most powerful tool for visualizing the nidus and adjacent osteosclerosis (Fig. 14).

**Fig. 15:**
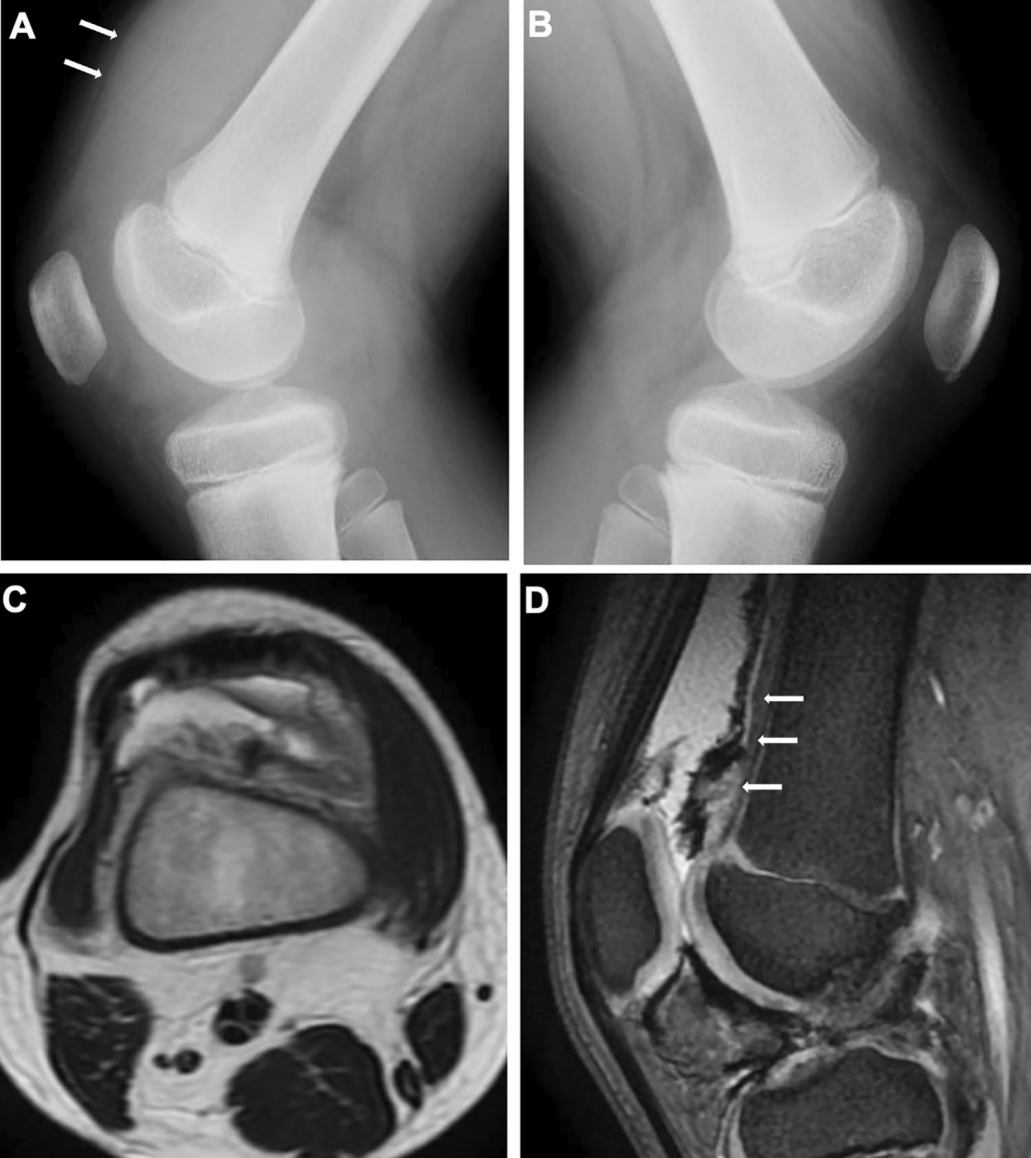
Tenosynovial giant cell tumor. **A–D**. A 9-year-old boy with swelling of the right knee. Lateral radiographs of the knees showed distention of the right suprapatellar bursa (arrows, **A**) and a normal left knee (**B**). Axial T2-weighted image (**C**) and sagittal gradient echo image (**D**) revealed synovial thickening and hemosiderin deposition lining the synovium. Hemosiderin is more clearly depicted on gradient echo image (arrows, **D**)

Tenosynovial giant cell tumor, formerly known as pigmented villonodular synovitis (PVNS), is a benign neoplasm involving the synovium, bursae, and tendon sheath. It is prevalent in the third and fourth decades of life but can occur at any age. PVNS is characterized by villous, nodular, synovial proliferation associated with hemosiderin-laden, multinucleated giant cells, and is divided into the diffuse and localized types. The former is more invasive, causing bone erosion and destruction. Identification of hemosiderin deposition in the proliferated synovium is essential for diagnosing PVNS. Susceptibility-sensitive imaging is most useful. “Blooming” of the susceptibility artifact is well demonstrated in gradient echo sequences (Fig. 15).

Hemophilic arthropathy results from recurrent joint bleeding in children with severe hemophilia A or B. Intra-articular hemorrhage occurs frequently in the large weight-bearing joints of the legs. Hemarthrosis may be the first symptom to come to medical attention. Repeated hemarthrosis leads to synovial hypertrophy and its destructive domino effect. The imaging findings of hemophilic arthropathy are indistinguishable from those of oligoarticular JIA on plain radiography. However, recurrent hemarthrosis gives rise to hemosiderin deposition in the synovium. MRI findings of hemophilic arthropathy are identical to those of diffuse type PVNS. Juxta-articular osteopenia is less conspicuous in either disorder than in JIA.

There are a number of disorders that should be differentiated from monoarticular and polyarticular involvement in JIA. The former is marked by transient synovitis of the hip and Legg-Calve-Perthes disease, while the latter is marked by leukemia. Imaging examinations are beneficial for the differential diagnosis of these disorders and JIA.

## Systemic JIA

There is a consensus that systemic JIA or systemic arthritis is a form of autoinflammatory disease; its pathogenesis involves the autoinflammatory mechanism (hyperactivity of the innate immune system) rather than the autoimmune mechanism (hyperactivity of the acquired immune system). The pathomechanism leads to excessive production of inflammatory cytokines, such as IL-6, IL-1β, and IL-18, and impairs the production of anti-inflammatory cytokines, such as IL-10. The pathogenesis of this disease is similar to that of adult-onset Still’s disease.

The diagnostic criteria of systemic arthritis comprise the presence of arthritis, characteristic fever, and extra-articular features. Affected children present with a high- spiking fever occurring on a daily or twice-daily basis and lasting at least for 2 weeks and have one or more of the following findings: transitory rash, generalized lymphadenopathy, hepatosplenomegaly, and serositis. Systemic and extra-articular illness may precede joint disease. The peak age at onset is 1–5 years. The female-to-male ratio is almost 1:1. Elevated IL-18 aids the diagnosis. Affected children are at risk of macrophage activation syndrome associated with life-threatening cytokine storm.

**Fig. 16:**
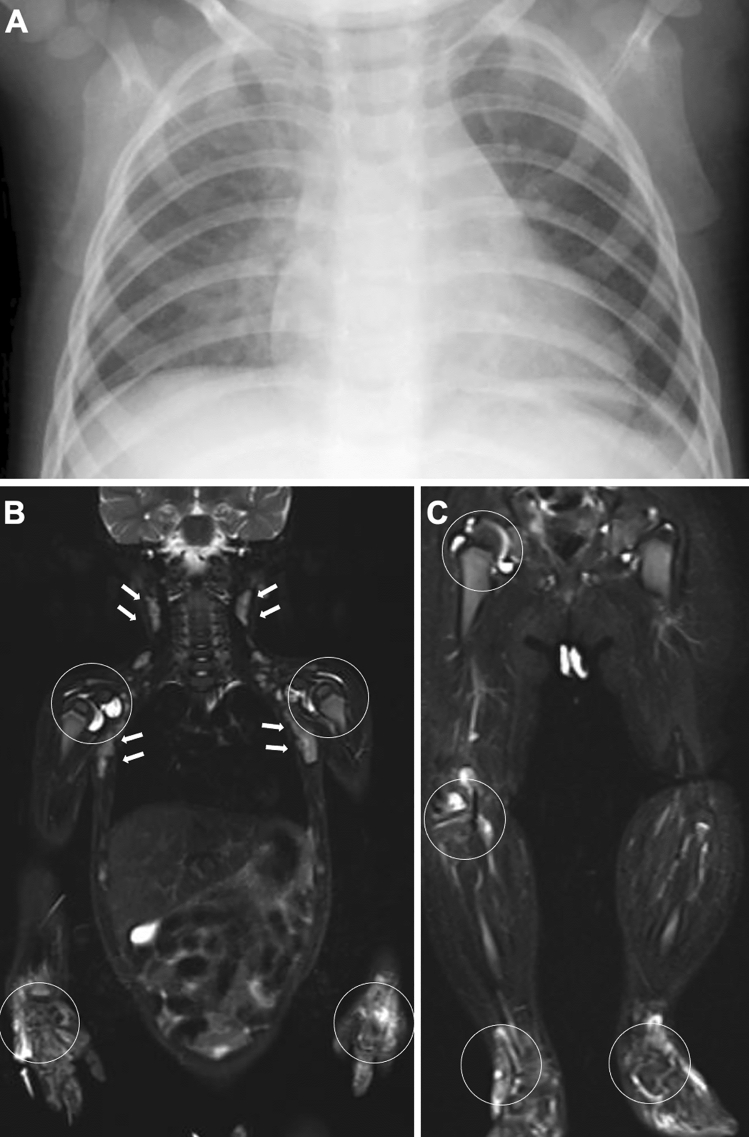
Systemic JIA. **A–C**. A 10-month-old boy with fever, rash, and multiple joint swelling. Frontal chest radiograph showed cardiac enlargement and mild pulmonary edema (**A**). Whole body STIR images (**B**, **C**) demonstrated bilateral symmetrical polyarthritis (increased joint fluid and edema of the carpal region) (circles, **B**, **C**) as well as cervicoaxillary lymphadenopathy (arrows, **B**)

**Fig. 17:**
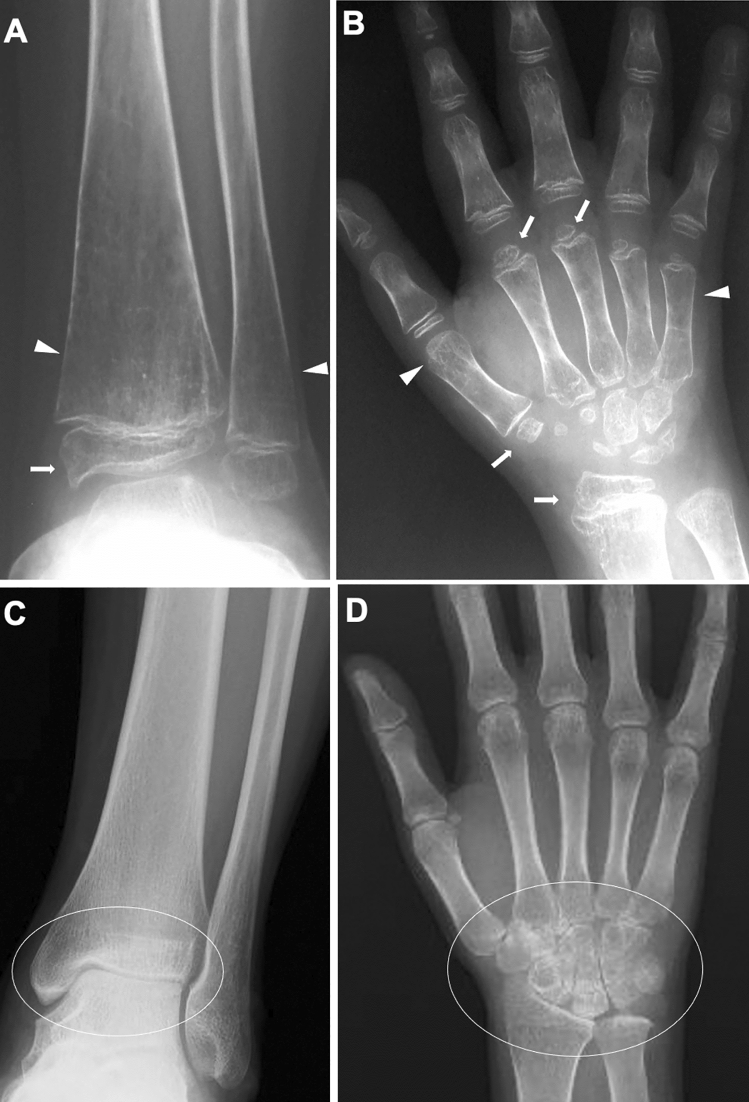
Different late sequelae between systemic JIA and polyarticular JIA. **A–B**. A 10-year-old boy with systemic JIA. Frontal radiographs of the left lower leg (**A**) and the right hand (**B**) show severe osteopenia, abnormal epiphyseal ossification (arrows, **A**, **B**), and metaphyseal broadening (arrowheads, **A**, **B**), but relatively spared joint spaces. **C–D**. A 16-year-old girl with polyarticular JIA. Frontal radiographs of the left lower leg (**C**) and the right hand (**D**) demonstrate striking joint space narrowing and minimal or mild subchondral bone irregularities (circles). Courtesy of Professor Yutaka Inaba, Department of Orthopedics, Yokohama City University Hospital

Arthritis can be monoarticular, but oligo- or polyarticular involvement is common. The pattern of the arthritis varies. Any large or small joint can be affected. Involvement of the cervical spine and TMJ is common. Tenosynovitis, enthesitis, and bursitis may occur. About 50% of affected children experience polyarthritis (Fig. 16). The prognosis is guarded. Approximately 25% of patients have severe physical morbidities related to the arthritis. Arthritis causes abnormalities of epiphyseal ossification, physeal endochondral growth, and modeling, which are more severe than in other types of JIA (Fig. 17). However, joint space narrowing is less conspicuous. Increased cytokines, particularly IL-6, IL-1, and TNFα, lead to severe osteoporosis. In fact, RANKL (a key molecule in osteoclastogenesis) is overexpressed in the synovium.

## Systemic autoinflammatory disease

Systemic autoinflammatory diseases encompass a group of disorders caused by aberrant activation of the innate immunity. This concept was introduced in 1999 by Kastner et al. as diseases with recurrent inflammatory episodes without infection or autoantibodies [24]. This group of disorders is either monogenic or polygenic. The former includes familial Mediterranean fever, NLRP3-associated autoinflammatory disease, tumor necrosis factor receptor-associated periodic syndrome, Blau syndrome (pediatric sarcoidosis), and Majeed syndrome, while the latter includes systemic JIA, adult-onset Still's disease, and chronic recurrent multifocal osteomyelitis (CRMO) [2–4, 25]. Common symptoms include intermittent fever, skin rash, eye symptoms, lymphadenopathy, and arthralgia. Diagnosis is often delayed, because the initial manifestations are non-specific. It is intriguing that the autoinflammatory mechanism plays a primary pathogenic role in a number of common diseases, such as Crohn’s disease and asbestosis. Addressed here are only a few systemic autoinflammatory disease with distinctive bone and joint abnormalities.

### NLRP3-associated autoinflammatory disease (NLRP3-AID)

NLRP3-AID, formerly termed cryopyrin-associated periodic syndrome (CAPS), comprises a group of disorders arising from monoallelic, gain-of-function mutations in the *NLRP3* (*CIAS1*) gene, which induce neutrophil-mediated inflammation [15]. NLRP3-AID comprises three subtypes of increasing severity, namely, familial cold autoinflammatory syndrome, Muckle-Wells syndrome, and CINCA (chronic infantile neurological cutaneous and articular syndrome) also known as NOMID (neonatal onset multisystem inflammatory disease). These are autosomal dominant disorders, but about a half of NOMID patients have somatic mosaic mutations that are difficult to confirm in blood samples. They have several features in common, including intermittent fever associated with increased acute reactants, neutrophilic urticaria-like skin rash, and joint involvement varying in severity. NOMID is associated with chronic aseptic meningitis, which causes a range of neurological deficits.

**Fig. 18:**
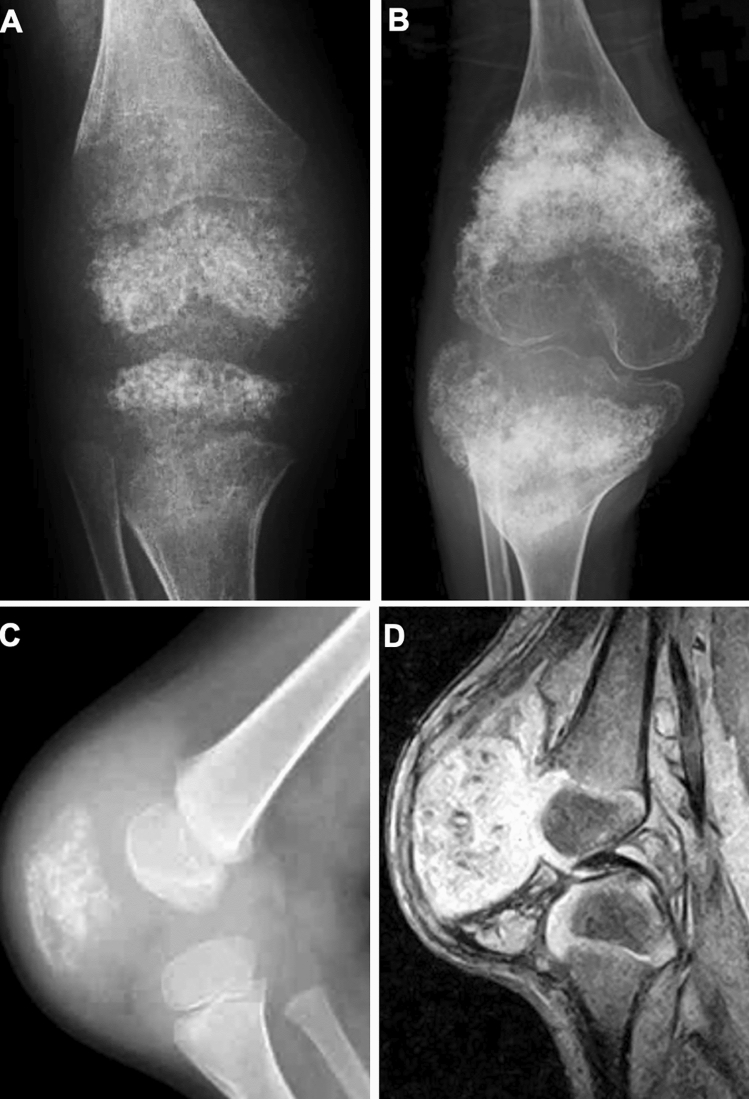
Two cases with CINCA. **A–B**. A child with a long history of systemic illness. The frontal radiographs of the knees show age-dependent evolution of epi-metaphyseal cartilaginous overgrowth with abnormal ossifications at age 2 years (**A**) and 14 years (**B**). **C**-**D**. A different child. The lateral radiograph of the knee shows characteristic patellar overgrowth with abnormal ossifications (**C**). Sagittal gradient echo image shows a markedly enlarged patellar cartilage with the same signal intensity as an articular cartilage, associated with low signal spots reflecting internal calcification (**D**)

Bone and joint involvement in CINCA is seen in the large joints and most often the knee [25]. Its hallmark is abnormal endochondral ossification leading to epimetaphyseal osteocartilaginous overgrowth resulting in bulbous joints associated with joint restriction. Patellar enlargement is the most characteristic presentation (Fig. 18). Epiphyseal enlargement is much severer than in JIA. The metaphyseal changes may resemble those in enchondromatosis (Ollier’s disease). The metaphyses can be bilaterally and symmetrically affected, the finding of which may lead to the misdiagnosis of metaphyseal chondrodysplasia. Synovitis is present but mild. Anakinra, an IL-1 receptor antagonist, is beneficial for relieving the systemic inflammation and inhibiting cartilage overgrowth [26, 27]. Mild patellar overgrowth may be seen in Muckle-Wells syndrome. Long-term, neutrophil-mediated, systemic inflammation in NLRP3-AID may cause secondary amyloidosis.

### Blau syndrome

**Fig. 19:**
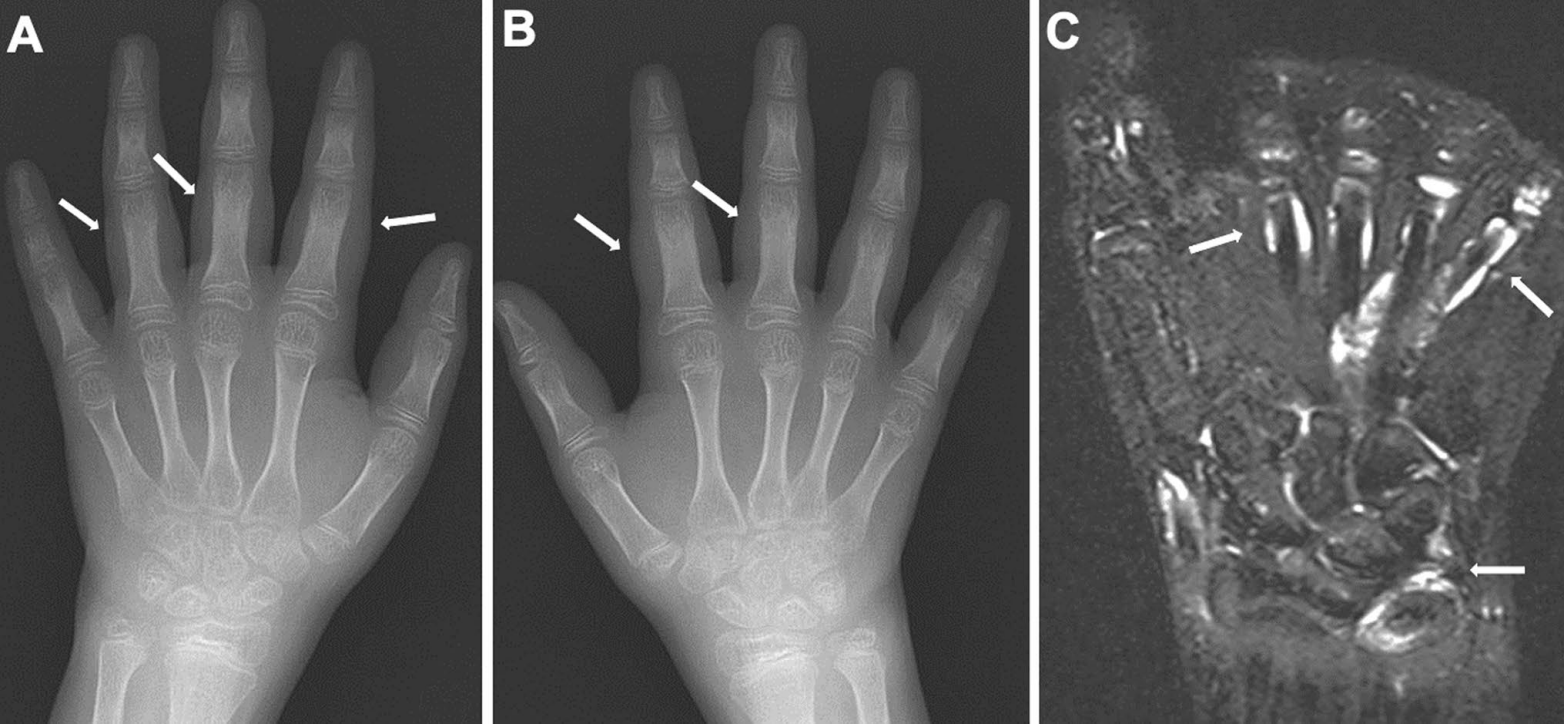
Blau syndrome. **A–C**. A 9-year-old girl with swelling of the hands. The frontal radiographs of the hands show bilateral, symmetric sausage-like swelling of the fingers (arrows, **A**, **B**). Periarticular osteopenia is also seen. Fat-suppressed T2-weighted coronal image shows fluid accumulation in the tendon sheath and intercarpal joints (arrows, **C**)

Blau syndrome is a granulomatous disorder that is histologically similar to, but clinically different from, adult sarcoidosis. It is characterized by a triad of polyarthritis and/or tenosynovitis, uveitis, and rash and is caused by gain-of-function mutations in the *NOD2* gene. The result is painless arthritis of the wrists, ankles, knees, and PIP joints as well as granulomatous tenosynovitis. The latter may manifest as a mass-like swelling on the dorsum of the hands and feet, called “boggy synovitis”. Tenosynovitis of the digits presents as sausage-like swelling of the fingers and toes (Fig. 19). Persistent tenosynovitis of the hands results in restriction and subluxation of the small joints.

The common radiological findings are progressive camptodactyly and carpal arthritis accompanied by symmetrical periarticular osteopenia and joint space narrowing but less conspicuous joint erosion even at the late stage. Radial and ulnar epimetaphyseal deformities are also characteristic [28]. The predominant carpal involvement mimics that of polyarticular JIA. In Blau syndrome, however, the affected joints are painlessness, tenosynovitis is more conspicuous than synovitis, the axial skeleton and TMJ are unaffected, and erosive changes are minimal [16]. Uveitis may threaten the quality of life of the patients.

### Chronic recurrent multifocal osteomyelitis (CRMO)

CRMO comprises heterogeneous forms of chronic, noninfectious, multifocal osteomyelitis stemming from the dysregulation of autoinflammatory cytokines. This group of disorders is divided into rare, hereditary (monogenic) diseases and more common, non-hereditary (multifactorial) diseases. The former comprises Majeed syndrome, DIRA (deficiency of IL-1 receptor antagonist), and PAPA (pyogenic arthritis, pyoderma gangrenosum, and acne) syndrome, which are characterized by early onset of bone changes and association with neutrophilic dermatosis, i.e., acute febrile neutrophilic dermatosis (Sweet syndrome) in Majeed syndrome, generalized pustulosis in DIRA, and sterile pyoderma in PAPA. Majeed syndrome is also associated with dyserythropoietic anemia. In particular, DIRA shows striking bone changes resembling those of NOMID/CINCA. On the other hand, non-hereditary CRMO is an indolent disorder, and it is thought to be a manifestation of pediatric SAPHO syndrome (synovitis, acne, pustulosis, hyperostosis, osteitis). Herein we focused on only the clinical and radiological features of non-hereditary CRMO.

Non-hereditary CRMO has its onset most commonly between ages 9 years and 14 years. As the disease name indicates, the bone changes have a relapsing and remitting course and may be clinically silent. However, the prolonged inflammation may impair normal growth of affected bones. Histological examination yields only nonspecific, culture-negative, chronic, inflammatory changes.

**Fig. 20:**
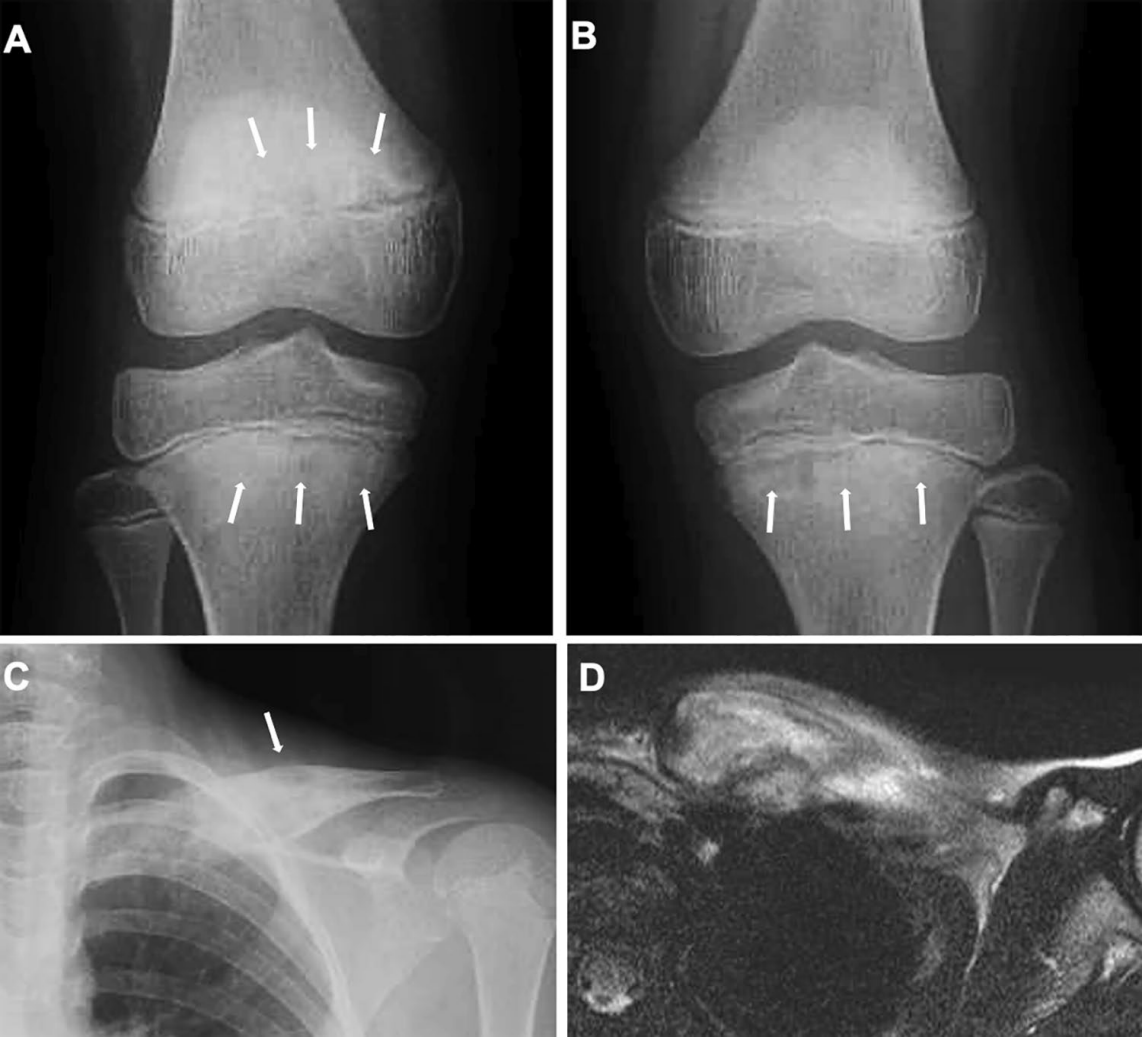
CRMO. **A–D**. A 9-year-old girl with recurrent left clavicular pain over several months and recent development of painful bilateral knees. The frontal radiographs of the knee demonstrate mixed sclerotic and lytic changes in the right distal femoral meteaphysis and bilateral proximal tibial metaphyses (arrows, **A**, **B**). The frontal radiograph of the left clavicle shows expansion and sclerosis of the medial and middle clavicular segments (arrow, **C**). Coronal fat-suppressed T2-weighted image reveals intraosseous high signal intensities of the thickened clavicle associated with surrounding soft tissue edema (**D**). There is no evidence of abscess formation

Bilateral symmetric, multifocal, metaphyseal osteomyelitis of the long bones is a classic manifestation (Fig. 20A, B). If the inflammation extends beyond the physis into the epiphysis, patients have a risk of growth impairment [29].The medial side of the clavicle, pelvic bones, and mandible are frequently affected (Fig. 20C, D) [30]. Bone changes often begin with “osteitis” that presents as the admixture of osteolytic foci and reactive sclerosis. Then, affected bones become expanded along with periosteal reaction or periosteal bone formation that is essentially smooth and uninterrupted. During regression, the bone changes are remodeled and normalized. The absence of intraosseous and/or subperiosteal abscesses is the rule [31], and MRI is useful for this evaluation, and also for assessing the activity of inflammation. Whole-body MRI and bone scintigraphy aid in diagnosing asymptomatic lesions [32].

## Conclusion

We reviewed the imaging characteristics of JIA. Since bone erosions occur later in JIA, aberrant epimetaphyseal growth often gives the first clue to the diagnosis. Oligoarthritis is the most common subtype of JIA, the insidious onset of which often delays the diagnosis and the monoarticular involvement should be distinguished from nonrheumatic arthropathies. Other types overlap with adult rheumatic diseases but possess unique characteristics. Systemic JIA is currently regarded as an autoinflammatory disease.
